# Efficacy and Safety of PD-1/PD-L1 Inhibitors Plus Chemotherapy Versus PD-1/PD-L1 Inhibitors in Advanced Non-Small Cell Lung Cancer: A Network Analysis of Randomized Controlled Trials

**DOI:** 10.3389/fonc.2020.574752

**Published:** 2021-01-11

**Authors:** Xiang Li, Shi Yan, Jichun Yang, Yaqi Wang, Chao Lv, Shaolei Li, Jun Zhao, Yue Yang, Minglei Zhuo, Nan Wu

**Affiliations:** ^1^Key Laboratory of Carcinogenesis and Translational Research (Ministry of Education), Department of Thoracic Surgery II, Peking University Cancer Hospital & Institute, Beijing, China; ^2^Central Laboratory, The Second Affiliated Hospital of Kunming Medical University, Kunming, China; ^3^Key Laboratory of Carcinogenesis and Translational Research (Ministry of Education), Department I of Thoracic Oncology, Peking University Cancer Hospital & Institute, Beijing, China

**Keywords:** PD-1/PD-L1 inhibitors, late-stage non-small cell lung cancer, PD-L1 expression level, survival efficacy, safety, first line treatment

## Abstract

Immune checkpoint inhibitors (ICIs) are recommended as first-line treatment for late-stage non-small cell lung cancer (NSCLC), either as monotherapy or in combination with chemotherapy. However, efficacy and safety comparisons between ICIs as monotherapy and ICIs with chemotherapy are lacking. We searched PubMed, Embase, and Cochrane Library for randomized controlled trials published before February 29th, 2020, with the search terms “immunotherapy” and “chemotherapy”. 10 eligible trials were identified with a total of 5,956 patients. Of these patients, 3,204 received immune therapy and 2,752 received chemotherapy. PD-1 inhibitors with chemotherapy improved OS (HR 0.84, 0.77–0.92), PFS (HR 0.80, 0.75–0.85), and objective response rate (ORR) (odds ratio (OR) 2.55, 1.20–5.28) compared to PD-1 inhibitors as monotherapy. In contrast, PD-L1 inhibitors plus chemotherapy showed no significant differences in OS, PFS, or ORR compared with PD-L1 inhibitors as monotherapy. When patients were stratified according to PD-L1 expression level, patients with high PD-L1 expression (≥ 50%) receiving PD-1 inhibitors plus chemotherapy had improved PFS, but not other outcomes, compared to PD-1 inhibitors as monotherapy. In these patients, PD-L1 inhibitors plus chemotherapy showed no significant difference in survival compared with PD-L1 inhibitors. In the low PD-L1 expression group (1%–49%), PD-1 inhibitors plus chemotherapy improved OS and PFS, but no advantage was observed in PD-L1 inhibitors plus chemotherapy in OS, PFS, or ORR compared with PD-L1 inhibitor monotherapy. When comparing PD-1/PD-L1 inhibitors plus chemotherapy with PD-1/PD-L1 inhibitors monotherapy, no significant differences were observed in the rate of immune-related adverse events (AEs). In summary, for treating patients with late-stage NSCLC, PD-1 inhibitors plus chemotherapy have improved efficacy compared with PD-1 inhibitor monotherapy, but PD-L1 inhibitors plus chemotherapy have similar efficacy as PD-L1 monotherapy. Survival benefits of PD-1/PD-L1 inhibitors combined with chemotherapy were particularly significant in patients with low PD-L1 expression levels.

**Systematic Review Registration:**

PROSPERO, identifier CRD42020166678 (https://www.crd.york.ac.uk/prospero/display_record.php?RecordID=166678).

## Introduction

Worldwide in 2018, 2,093,876 new lung cancer patients were diagnosed and lung cancer caused 1,761,007 deaths (1). Non-small cell lung cancer (NSCLC) accounts for 80%–85% of these cases (2). Therapeutic regimes for patients with NSCLC in stage III or beyond include radical radiotherapy, chemo-radiotherapy, gene targeted therapy, and immune checkpoint inhibitors (ICIs).

ICI therapies, targeting T-cell regulatory pathways to provide significant clinical benefits against cancers (3, 4), have been heralded as a promising treatment for lung cancer. The receptor PD-1 and its ligands PD-L1 and PD-L2 play a vital role in the maintenance of immunologic self-tolerance (5). Cancers can exploit this pathway to escape T-cell-mediated attack by the immune system. Clinical practice has attempted to enhance anti-tumor immune responses by augmenting costimulatory signals, but coinhibitory signals that block anti-tumor T-cell responses have been shown to be more effective than costimulatory signals (6).

MDX1105 (nivolumab) was the first PD-1 inhibitor used in cancer therapy. In 2010, it was reported that one patient with colorectal cancer achieved a complete response and two patients with melanoma and renal cancer achieved partial response (7). More recent data have indicated that 18% of NSCLC patients respond to treatment with MDX11105 (8).

NCCN guidelines recommend the PD-1 inhibitor pembrolizumab as first-line treatment for NSCLC with PD-L1 expression level ≥50%. KEYNOTE-024 (9) demonstrated that pembrolizumab improved median OS compared to chemotherapy, with OS of 30 months with pembrolizumab and 14.2 months with chemotherapy. Similarly, KEYNOTE-042 (10) showed that median OS was 22.3 months in patients treated with pembrolizumab and 10.5 months in patients treated with chemotherapy. KEYNOTE-407 (11) evaluated pembrolizumab plus chemotherapy in patients with stage IV squamous NSCLC. In this study, median OS was 15.9 months in patients receiving combination therapy and 11.3 months in patients receiving chemotherapy. A similar trend was observed in non-squamous NSCLC patients in IMpower 130, in which the median OS was 18.6 months in patients receiving the PD-L1 inhibitor atezolizumab plus chemotherapy and 13.9 months in patients receiving chemotherapy. Comparisons between PD-1/PD-L1 inhibitor monotherapy and chemotherapy confirmed the survival benefits of immunotherapy (9, 10, 12, 13). The NCCN NSCLC panel recommended single-agent pembrolizumab as first-line therapy for NSCLC patients with PD-L1 expression level >1%, and ICIs plus chemotherapy were recommended for patients who could tolerate adverse events (AEs) (14, 15).

Although immunotherapy has gradually become the mainstay in NSCLC therapy, optimization of the treatment plan for advanced NSCLC patients is still facing challenge. This network analysis aims to compare the efficacy and safety of PD-1/PD-L1 inhibitors plus chemotherapy with PD-1/PD-L1 monotherapy.

## Methods

### Data Sources and Searches

This network analysis was conducted using Preferred Reporting Item for Systemic Reviews and Meta-Analyses guidelines (16). The review is registered on the PROSPERO website as No. CRD42020166678. PubMed, Embase, and Cochrane Library were systematically searched up to February 29^th^, 2020, using the search terms “non-small cell lung cancer,” “immune checkpoint inhibitors,” “immune checkpoint blockade,” “pembrolizumab,” “nivolumab,” “atezolizumab,” “durvalumab,” and “chemotherapy.” See **Supplementary Material** for more information.

### Study Selection

Two reviewers (XL and SY) independently screened the study titles and abstracts based on predefined inclusion and exclusion criteria. The reviewers discussed any discrepancies or, if necessary, by seeking a decision from a third reviewer (NW).

Inclusion criteria for studies were as follows: (i) Patients had histologically confirmed previously untreated unresectable advanced (stage IIIB/IIIC/IV) NSCLC without *EGFR* or *ALK* mutations. (ii) Interventions were PD-1/PD-L1 inhibitor plus platinum-based chemotherapy or PD-1/PD-L1 inhibitor alone. (iii) Comparators were platinum-based chemotherapy. (iv) Outcomes were efficacy outcomes, including OS, PFS, ORR; and safety outcomes, including AEs. OS was defined as the time from when patients enrolled into trials to death with any cause. PFS was defined as the time from randomization until progression for any cause. ORR was defined as the proportion of patients achieving partial or complete remission. AEs were defined and graded according to the common terminology criteria from the National Cancer Institute (17). (v) Only randomized controlled trials (RCTs) were included.

Studies were excluded according to the following exclusion criteria: (i) Patients had cytologically or histologically confirmed small cell lung cancer or other kinds of lung cancer. Patients with driver gene-mutations were excluded. Patients who had received previous systemic treatment were excluded. (ii) Patients who received spontaneous anti-CTLA-4 were excluded. Patients who received concurrent or sequential radio-chemotherapy were excluded. (iii) Studies other than RCTs were excluded.

### Data Extraction and Risk of Bias Assessment

Data were extracted using ADDIS 16.7 software. Two investigators independently reviewed the full text of included studies and extracted information, including first author, year of publication, patient characteristics, inclusion and exclusion criteria, treatment protocol, outcomes, HR for OS, HR for PFS, OR for ORR, and OR for AEs. We concentrated on treatment-related severe AEs, defined as grades 3–5. The included trials were performed at multiple sites worldwide over long periods, so we extracted data from the most recent published articles or conference reports possible. Risk of bias of trials was assessed independently by two investigators using the Cochrane risk of bias tool (18). Differences in data extraction were mediated by Prof. N.W.

### Data Synthesis and Statistical Analysis

#### Direct Comparison

Pooled HRs with 95% confidence intervals (95% CI) were calculated for OS and PFS and pooled ORs with 95% CI were calculated for ORR and the rate of AEs using the random-effects model in REVMAN 5.3 (Cochrane). The quantity I^2^ was used to describe heterogeneity between studies. We included only low risk of bias in the sensitivity analysis.

#### Indirect and Mixed Comparisons

A random-effects network meta-analysis (NMA) within a Bayesian framework was then performed using OpenBUGS version 3.2.3. Pooled HRs and ORs with 95% CI were also summarized. Each treatment was ranked using the surface under the cumulative ranking curve (SUCRA) and a treatment hierarchy was generated. A treatment ranked as 100% is certain to be the best and a treatment ranked as 0% is certain to be the worst in terms of efficacy and safety outcomes. We also used the contribution plot to measure the percent contribution of each direct comparison to the mixed estimates, the indirect estimates, and the entire network, as shown in the **Supplementary Material**.

#### Examination of Assumptions in Network Meta-Analysis

To check the consistency of the NMA, we used the node-splitting model to assess inconsistencies between direct and indirect treatment effects. A predictive interval plot was used to estimate heterogeneity for all comparisons.

#### Additional Analyses

We used the comparison-adjusted funnel plot to assess small-study effects. The synthesized endpoints included OS, PFS, ORR, and treatment-related grade 3–5 AEs. HRs of OS and PFS were preferentially reported and were adjusted for confounders in individual studies (19). HR could also be estimated according to the method described by Tierney and colleagues (20). Consistency was assessed by comparing synthesized HR of NMA with pairwise head-to-head meta-analyses. The ORs of ORR and AEs were calculated using Bayesian statistics.

Bayesian NMA was done using a Markov Chain Monte Carlo simulation technique in OpenBUGS version 3.2.3. We used non-informative uniform and normal prior distributions (21) and three different sets of initial values to fit the model. For OS and PFS, 30,000 sample iterations were generated with 5,000 burn-ins and a thinning interval of 10. For ORR and AE, 50,000 sample iterations were generated with 20,000 burn-ins and a thinning interval of 10.

Transitivity was estimated by assessing studies that compared two treatments and evaluating direct and indirect comparisons (22). All included studies were RCTs that compared experimental treatments with platinum-based chemotherapy, providing convincing and stable transitivity.

Patients were stratified according to PD-L1 expression (≥ 50% or 1%–49%). PD-1 inhibitors and PD-L1 inhibitors were evaluated to determine their effect on the NMA.

## Results

### Systematic Review and Characteristics

**Figure 1:**
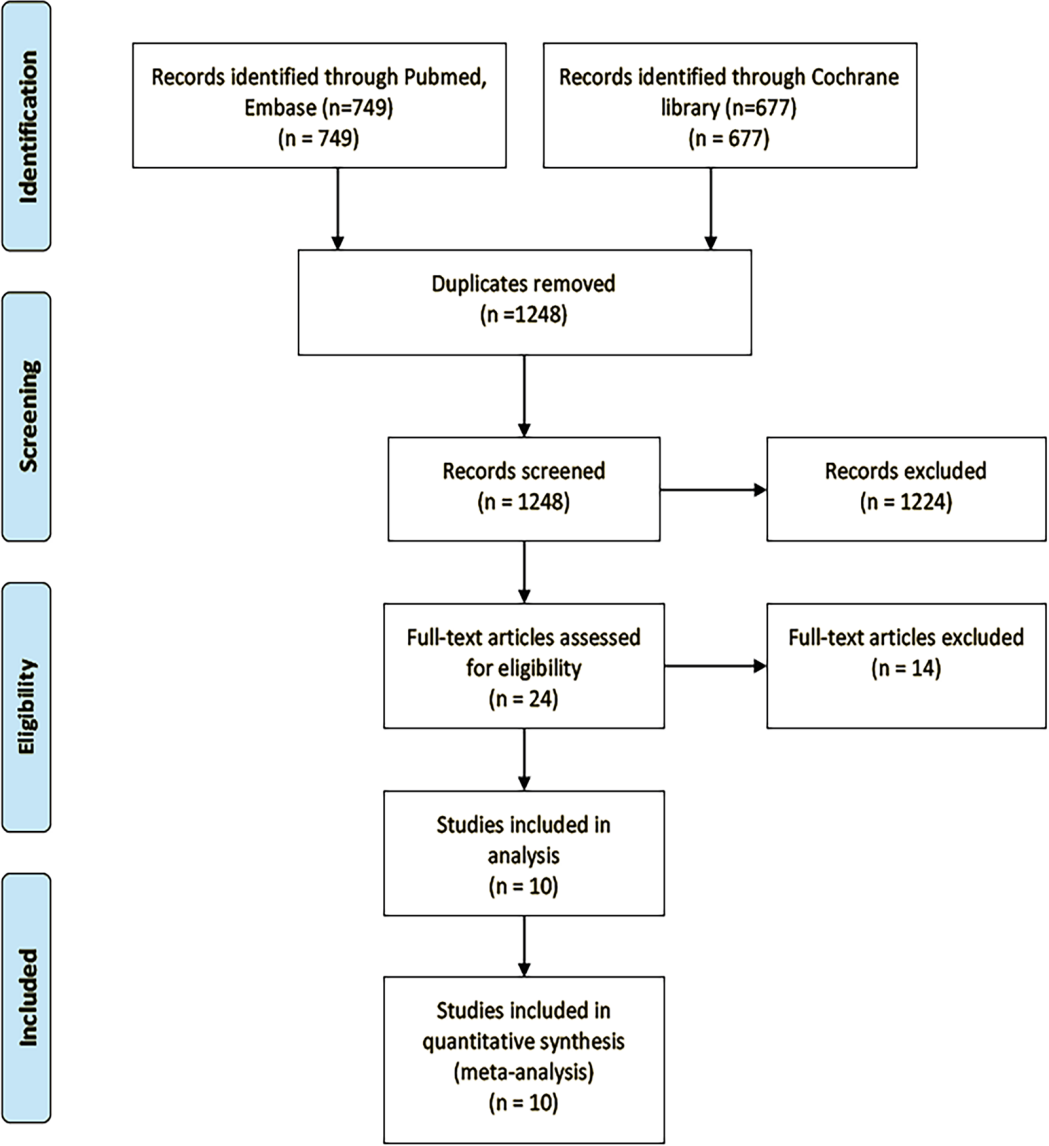
The flow diagram of studies selection.

We screened 1,426 records (**Figure 1**) and identified 24 studies for full-text reads. Ten eligible RCTs were included in this NMA (**Table 1**), with a total of 5,956 patients receiving one of six treatment strategies as first-line therapy for advanced unresectable lung cancer. Of these patients, 3,204 patients received immune therapy and 2,752 patients received chemotherapy. Most of the included trials were published with low bias (see **Supplementary Material**).

**Table 1:** Characteristics and results of included trials.

Patient Numbers		HR (95% CI)		ORR (%)	AEs	
		OS	PFS		All	Severe
**IMpower-110**	572					
Atezolizumab 1200 mg Q3W	286	0.83 (0.65–1.07)	0.77 (0.63–0.94)	29.2	258	91
Cisplatin 75 mg/m^2^ or carboplatin AUC 6, pemetrexed 500 mg/m^2^ IV Q3W; cisplatin 75 mg/m^2^ + gemcitabine 1,250 mg/m^2^ or carboplatin AUC 5 + gemcitabine 1,000 mg/m^2^ IV Q3W	286			31.8	249	144
**IMpower-130**	705					
Atezolizumab 1,200 mg Q3W, then carboplatin 6 mg/ml Q3W plus nab-paclitaxel 100 mg/m2 QW	473	0.79 (0.64–0.98)	0.64 (0.54–0.77)	49.2	455	354
Carboplatin 6 mg/mL Q3W + nab-paclitaxel 100 mg/m2 QW	232			31.9	215	141
**IMpower-131**	683					
Atezolizumab 1,200 mg IV Q3W, then carboplatin AUC 6 IV Q3W, nab-paclitaxel 100 mg/m^2^ IV QW	343	0.96 (0.78–1.18)	0.61 (0.48–0.77)	49	332	274
Carboplatin AUC 6 IV Q3W, nab-paclitaxel 100 mg/m^2^ IV QW	340			41	324	234
**IMpower-132**	578					
Atezolizumab 1,200 mg IV Q3W, then carboplatin AUC 6 mg/ml/min IV Q3W or cisplatin 75 mg/m^2^ IV Q3W, pemetrexed 500 mg/m^2^ IV Q3W	292	0.81 (0.64–1.03)	0.60 (0.49–0.72)	47	286	202
Carboplatin AUC 6 mg/ml/min IV Q3W or cisplatin 75 mg/m^2^ IV Q3W, pemetrexed 500 mg/m^2^ IV Q3W	286			32	266	161
**CheckMate-026**	541					
Nivolumab 3 mg/kg Q2W	271	1.02 (0.8–1.3)	1.15 (0.91–1.45)	26	190	47
Cisplatin 75 mg/m^2^ + gemcitabine 1,250 mg/m^2^ or carboplatin AUC 5 + gemcitabine 1,000 mg/m^2^ or carboplatin AUC 6 + paclitaxel 2,000 mg/m^2^; cisplatin 75 mg/m^2^ or carboplatin AUC 6 + pemetrexed 500 mg/m^2^	270			33	243	133
**KEYNOTE-021**	123					
Pembrolizumab 200 mg + carboplatin AUC 5 + pemetrexed 500 mg/m^2^ IV Q3W	60	0.90 (0.42–1.91)	1.17 (0.95–1.43)	42.7	55	23
Carboplatin AUC 5 + pemetrexed 500 mg/m^2^ IV Q3W	63			18.4	56	16
**KEYNOTE-024**	305					
Pembrolizumab 200 mg Q3W, 35 cycles	154	0.60 (0.41–0.89)	0.50 (0.37–0.68)	44.8	113	41
Carboplatin AUC 5/6 Q3W or cisplatin 75 mg/m^2^ + pemetrexed 500 mg/m^2^ Q3W; cisplatin 75 mg/m^2^ or carboplatin AUC 5/6 + gemcitabine 1,250 mg/m^2^ Q3W or paclitaxel 200 mg/m^2^ Q3W	151			41.98	135	80
**KEYNOTE-042**	1274					
Pembrolizumab 200 mg Q3W, 35 cycles	637	0.79 (0.64–0.98)	0.64 (0.54–0.77)	27	399	113
Carboplatin AUC 5/6 Q3W + paclitaxel 200 mg/m^2^ Q3W or carboplatin AUC 5/6 Q3W + pemetrexed 500 mg/m^2^ Q3W, 6 cycles	637			27	553	252
**KEYNOTE-189**	616					
Pembrolizumab 200 mg + pemetrexed 500 mg/m^2^ + carboplatin AUC 5 or cisplatin 75 mg/m^2^ Q3W, 4 cycles	410	0.79 (0.64–0.98)	0.64 (0.54–0.77)	47.6	404	272
Placebo (saline) + pemetrexed 500 mg/m^2^ + carboplatin AUC 5 or cisplatin 75 mg/m^2^ Q3W, 4 cycles	206			18.9	200	133
**KEYNOTE-407**	559					
Pembrolizumab 200 mg Q3W + carboplatin AUC 6 + paclitaxel 200 mg/m^2^ or nab-paclitaxel 100 mg/m^2^ Q3W, then pembrolizumab 200 mg Q3W	278	0.64 (0.49–0.85)	0.56 (0.45–0.70)	57.4 58.7	273	194
Carboplatin AUC 6 + paclitaxel 200 mg/m^2^ or nab-paclitaxel 100 mg/m^2^ Q3W, then pembrolizumab 200 mg Q3W	281			37.7 39.5	274	191

OS = overall survival; PFS = progression-free survival; ORR = objective response rate; AEs = adverse events; all = all AEs; severe = grade3-5 AEs.

**Figure 2:**
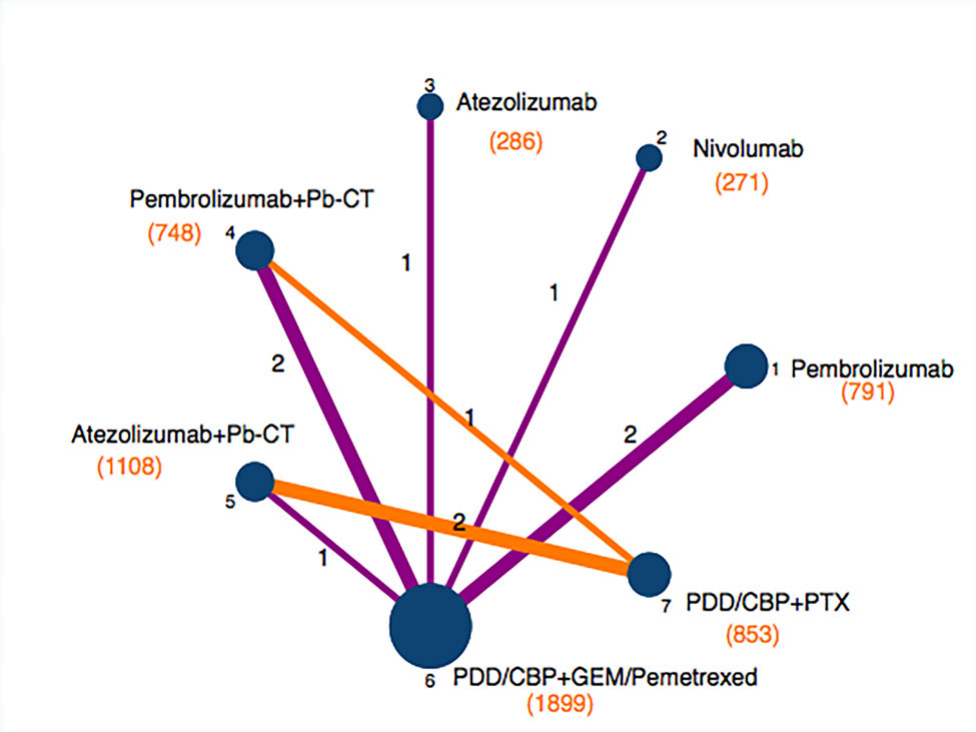
Net plot of included trials.

The included trials (9–13, 23–27) reported HRs for OS and PFS. Four of the trials (9, 10, 12, 13) evaluated a PD-1 or PD-L1 inhibitor as monotherapy while six of the trials (11, 23–27) evaluated a PD-1 or PD-L1 inhibitor in combination with chemotherapy. Six trials (9, 12, 13, 23, 24, 26) used a platinum-based chemotherapy plus pemetrexed or gemcitabine, three trials (11, 25, 27) used a platinum-based chemotherapy plus paclitaxel, and one trial (10) used a platinum-based chemotherapy plus pemetrexed/paclitaxel (**Table 1**). Some studies reported efficacy outcomes stratified according to PD-L1 expression; eight trials (9–13, 23, 25, 27) reported survival data in patients with high PD-L1 expression (≥ 50%) and five trials (10, 11, 23, 25, 27) reported survival data in patients with low PD-L1 expression (1-49%) (**Figure 2**).

### Results of Pairwise Meta-Analysis

Head-to-head comparisons revealed that compared with chemotherapy, OS was improved in patients treated with PD-1 inhibitors (HR 0.85, 0.76–0.95), PD-1 inhibitors plus chemotherapy (HR 0.57, 0.48–0.69), and PD-L1 inhibitors plus chemotherapy (HR 0.83, 0.74–0.94). PFS was also improved in patients treated with PD-L1 inhibitors (HR 0.77, 0.63-0.94), PD-1 inhibitors plus chemotherapy (HR 0.54, 0.47–0.62), and PD-L1 inhibitors plus chemotherapy (HR 0.65, 0.59–0.72). No significant difference in PFS was observed when comparing PD-1 inhibitors as monotherapy with chemotherapy (HR 1.0, 0.91–1.1).

### Efficacy Outcomes

Compared with PD-1/PD-L1 inhibitors as monotherapy, PD-1 and PD-L1 inhibitors combined with chemotherapy did not significantly improve OS (HR 0.94, 0.90–1.01), but the combination therapy did significantly improve PFS (HR 0.82, 0.78–0.87).

Compared with PD-1 inhibitor monotherapy, PD-1 inhibitors plus chemotherapy significantly improved OS (HR 0.84, 0.77–0.92), PFS (HR 0.80, 0.75–0.85), and ORR (OR 2.55, 1.20–5.28).

**Figure 3:**
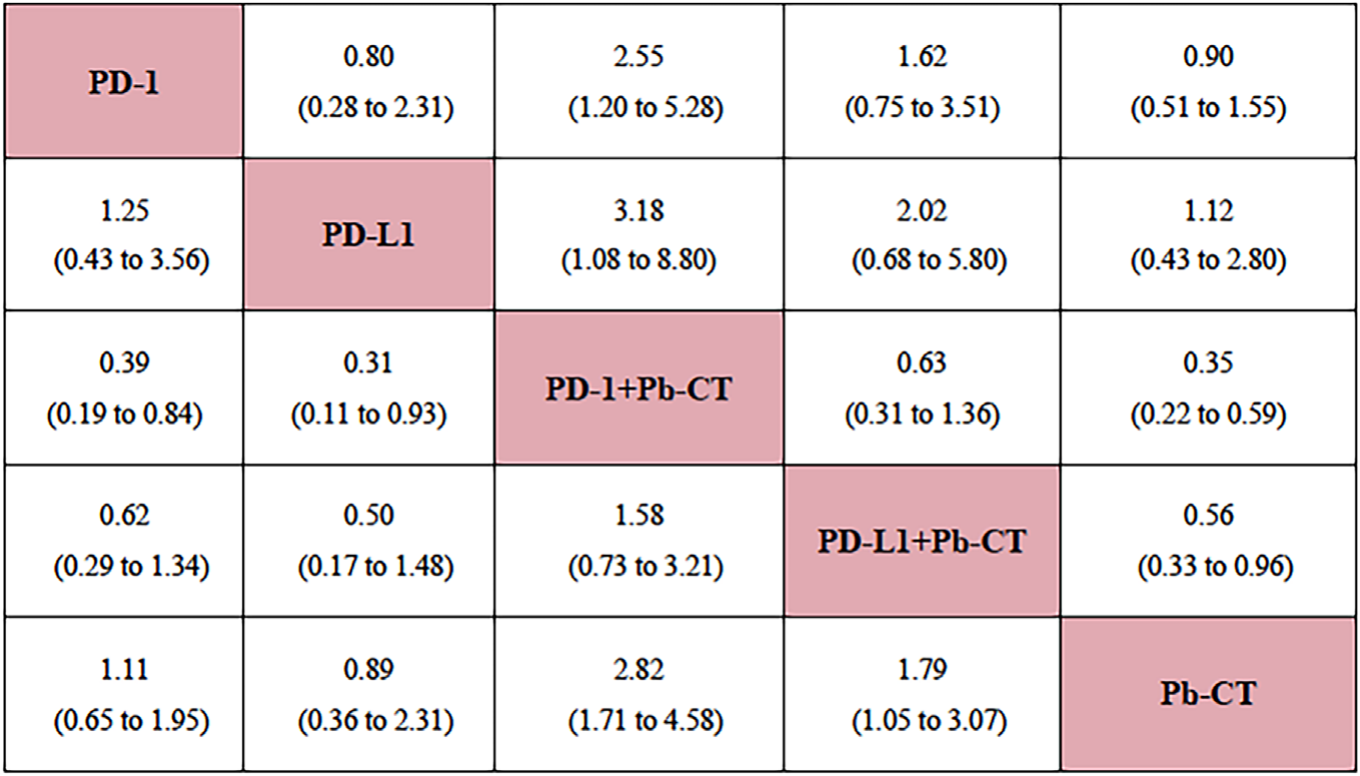
Network meta-analysis of objective response rate in all patients.

Compared with PD-L1 inhibitor monotherapy, PD-L1 inhibitors plus chemotherapy showed no significant difference in OS (HR 1.01, 0.89–1.13), PFS (0.91, 0.82–1.01), and ORR (OR 2.02, 0.68–5.80) (**Figure 3**).

**Figure 4:**
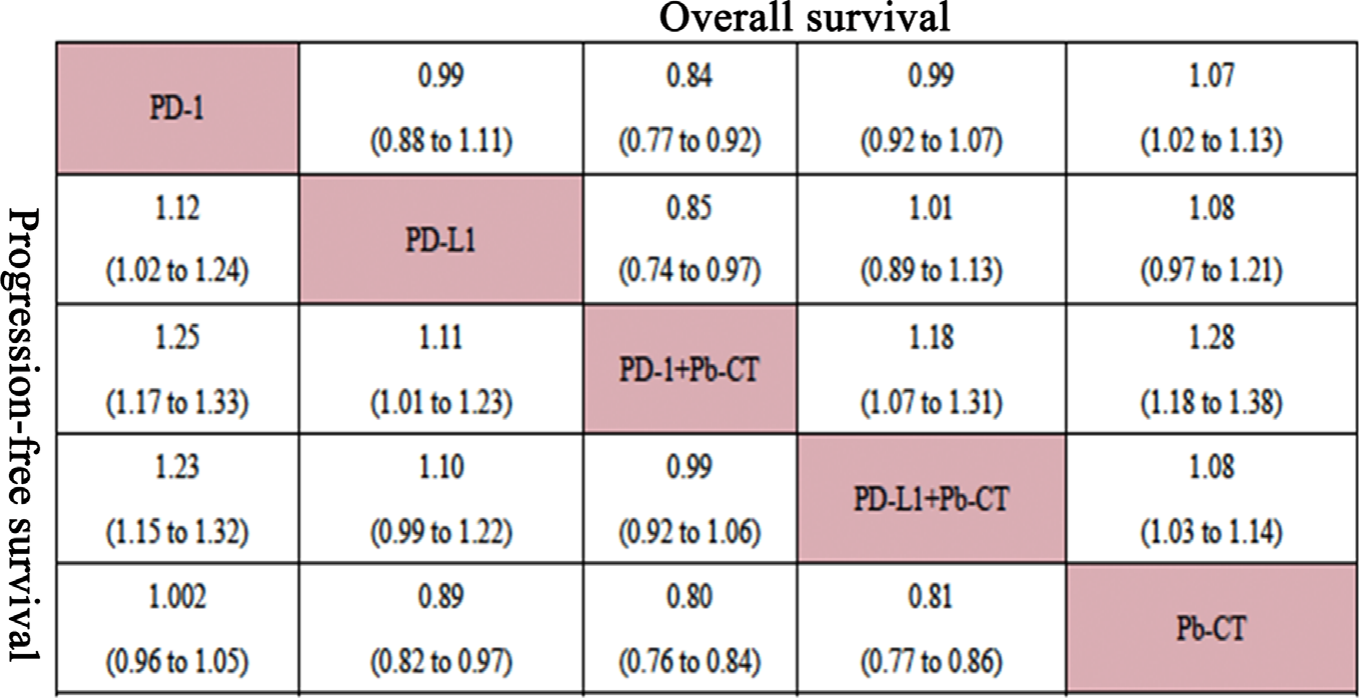
Network meta-analysis of all patients.

Compared with PD-L1 inhibitor plus chemotherapy, PD-1 inhibitor plus chemotherapy significantly improved OS (HR 0.85, 0.76–0.93) but not PFS (HR 0.99, 0.92–1.06) (**Figure 4**).

#### High PD-L1 Expression Level

In patients with high PD-L1 expression levels (≥ 50%), PD-1/PD-L1 inhibitors plus chemotherapy did not significantly improve OS compared to PD-1/PD-L1 inhibitors as monotherapy (HR 0.94, 0.82–1.07), but did significantly improve PFS (HR 0.78, 0.70–0.86). PD-1 inhibitors plus chemotherapy did not significantly improve OS compared with PD-1 inhibitors (HR 0.86, 0.73–1.03), but did significantly improve PFS (HR 0.72, 0.63–0.83). PD-L1 inhibitors plus chemotherapy did not significantly improve OS (HR 1.08, 0.85–1.37) or PFS (HR 0.89, 0.73–1.08) compared with PD-L1 inhibitors as monotherapy. PD-1 inhibitors plus chemotherapy significantly improved OS (HR 0.85, 0.76–0.93), but not PFS (HR 0.99, 0.92–1.06) compared with PD-L1 inhibitors plus chemotherapy.

#### Low PD-L1 Expression Level

**Figure 5:**
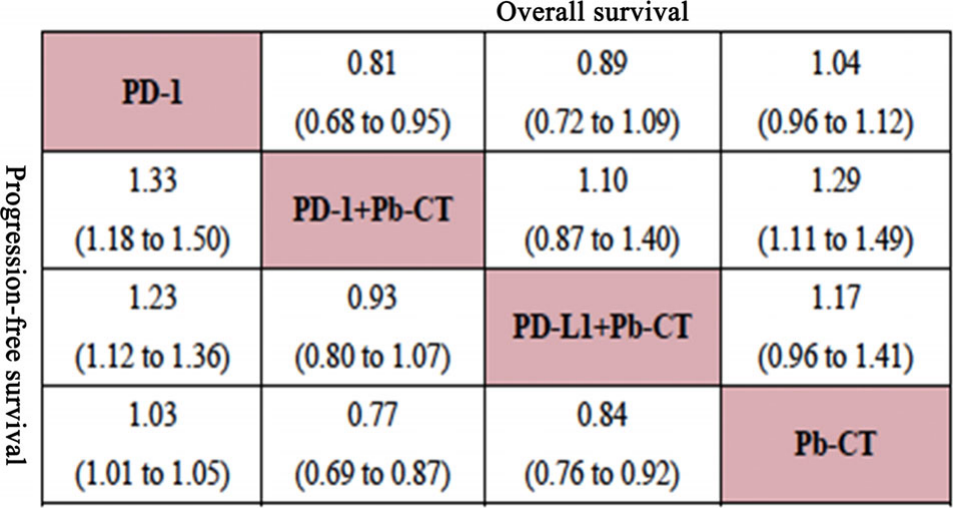
Network meta-analysis of patients with PD-L1 expression level 1% to 49%.

In patients with low PD-L1 expression levels (1%–49%), PD-1/PD-L1 inhibitors plus chemotherapy significantly improved OS (HR 0.84, 0.73–0.96) and PFS (HR 0.79, 0.73–0.85) compared with PD-1/PD-L1 inhibitors as monotherapy. PD-1 inhibitors combined with chemotherapy significantly improved OS (HR 0.81, 0.68–0.95) and PFS (HR 0.75, 0.67–0.85) compared with PD-1 inhibitors as monotherapy. No significant differences were observed in OS (HR 0.91, 0.71–1.15) or PFS (HR 0.93, 0.80–1.07) when comparing PD-1 inhibitors plus chemotherapy with PD-L1 inhibitors plus chemotherapy (**Figure 5**).

### Safety Outcomes

**Figure 6:**
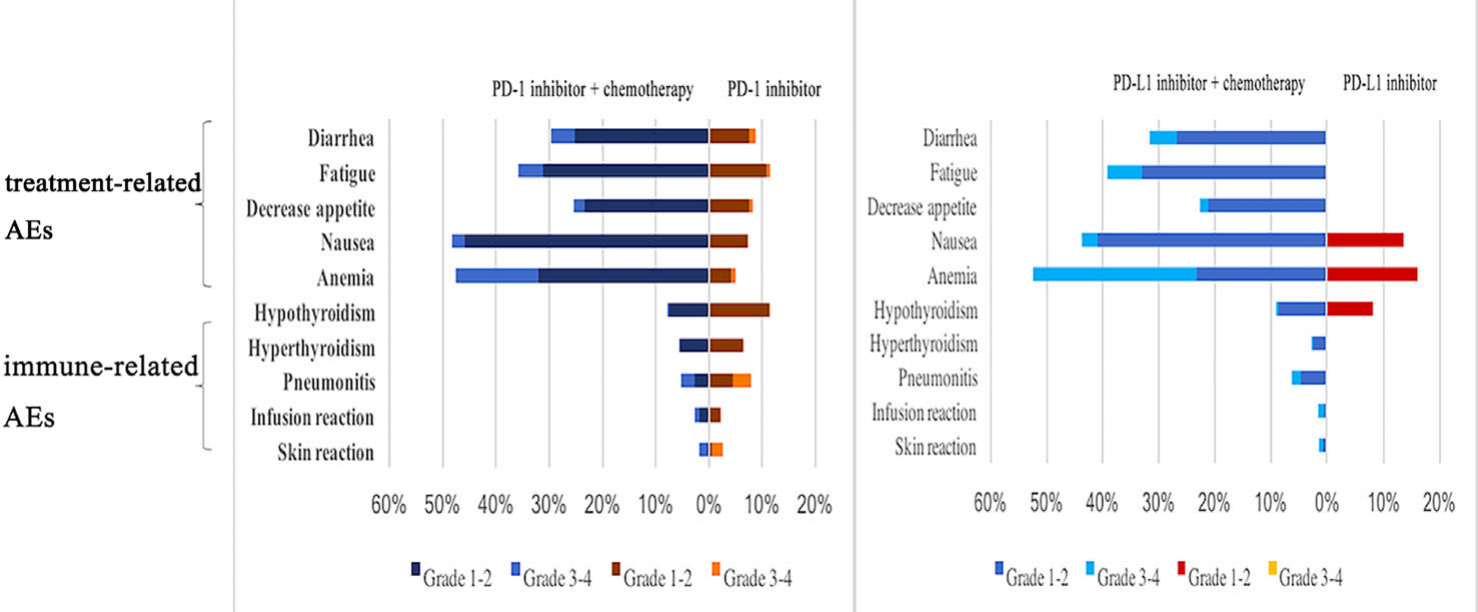
Proportion of adverse events in all patients.

**Figure 7:**
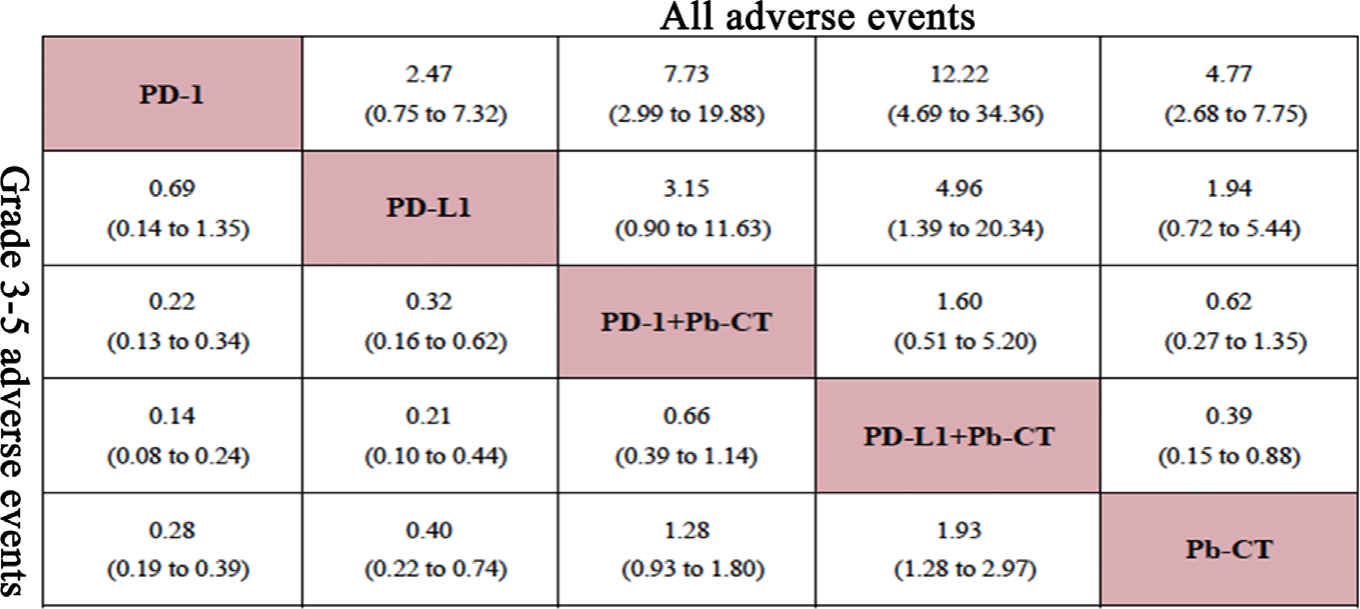
Network meta-analysis of adverse events in all patients.

In this network analysis, PD-1/PD-L1 inhibitors combined with chemotherapy were more likely to cause AEs, and especially severe AEs (grade 3–5) than PD-1/PD-L1 inhibitors as monotherapy (**Figure 6**). PD-1 inhibitors plus chemotherapy caused more AEs than PD-1 inhibitors as monotherapy when considering any AEs (OR 7.73, 2.99–19.88) and also when considering only severe AEs (OR 4.55, 2.94–7.69). Compared with PD-L1 inhibitor monotherapy, PD-L1 inhibitor plus chemotherapy caused more AEs overall (OR 4.96, 1.39–20.34) and more severe AEs (OR 4.76, 2.27–10.0). Compared with PD-1 inhibitor plus chemotherapy, PD-L1 inhibitor plus chemotherapy caused more AEs overall (OR 1.60, 0.51–5.20) and more severe AEs (OR 1.52, 0.88–2.56), but no significant differences were observed. Compared with PD-L1 inhibitors as monotherapy, PD-1 inhibitors as monotherapy did not cause significantly more overall AEs (OR 0.69, 0.14–1.35) or severe AEs (grade 3–5) (OR 0.41, 0.14–1.33) (**Figure 7**).

When comparing PD-1 inhibitors plus chemotherapy with PD-1 inhibitors as monotherapy, no significant differences were observed in the rate of immune-related AEs, including hypothyroidism (OR 0.41, 0.12–1.46), hyperthyroidism (OR 0.38, 0.04–3.75), pneumonia (OR 0.18, 0.03–1.41), and skin reactions (OR 0.10, 0.00–1.45). When comparing PD-L1 plus chemotherapy with PD-1 plus chemotherapy, no significant differences were observed in the rate of immune-related AEs, including hypothyroidism (OR 3.57, 0.68–24.47), hyperthyroidism (OR 4.82, 0.19–291.1), and pneumonia (OR 1.17, 0.17–13.44).

### Rank Probabilities

Ranking probabilities of the six treatments were summarized for OS, PFS, ORR, and AEs (**Supplementary Material**). PD-1 inhibitor plus chemotherapy provided the most favorable balance between efficacy and safety. For ORR, PD-1 inhibitor plus chemotherapy ranked first (89%) and PD-L1 inhibitor plus chemotherapy ranked second (77%). Similarly, PD-1 inhibitor plus chemotherapy had the highest SUCRA ranking. Nivolumab had the highest SUCRA ranking for AEs, causing the fewest severe AEs (98.9%) and overall AEs (90.5%). Pembrolizumab plus chemotherapy ranked first in OS (98.9%) and PFS (98.4%), pembrolizumab ranked second in OS (52.5%), and atezolizumab plus chemotherapy ranked second (91.2%) in PFS.

### Assessment of Heterogeneity

Heterogeneity estimates were calculated in four sub-group pairwise analyses (see **Supplementary Figures 8**, **9**, **40**). During analysis, no heterogeneity (I^2^ = 0%) or low heterogeneity (I^2^<50%) was used to assess comparisons. Notably, the I^2^ values of “PD-1 inhibitors versus platinum-based chemotherapy” were 75% for OS, 91% for PFS, and 33% for severe AEs. Meanwhile, the I^2^ value of “PD-1 inhibitors plus platinum-based chemotherapy versus platinum-based chemotherapy” showed moderated heterogeneity (41%). Other comparisons showed minimal heterogeneity (I^2 =^ 0%).

**Figure 8:**
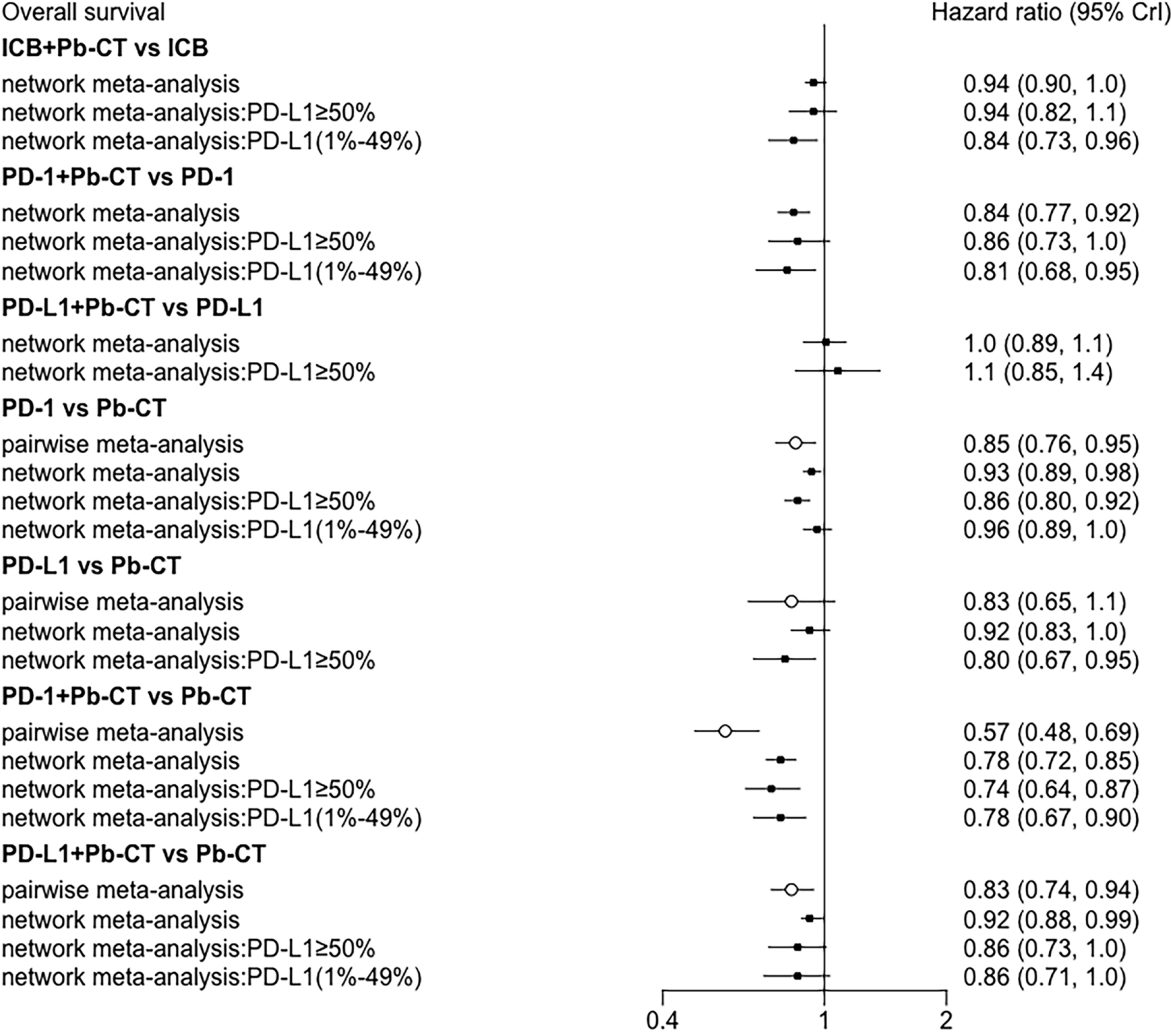
Consistency analysis for overall survival.

**Figure 9:**
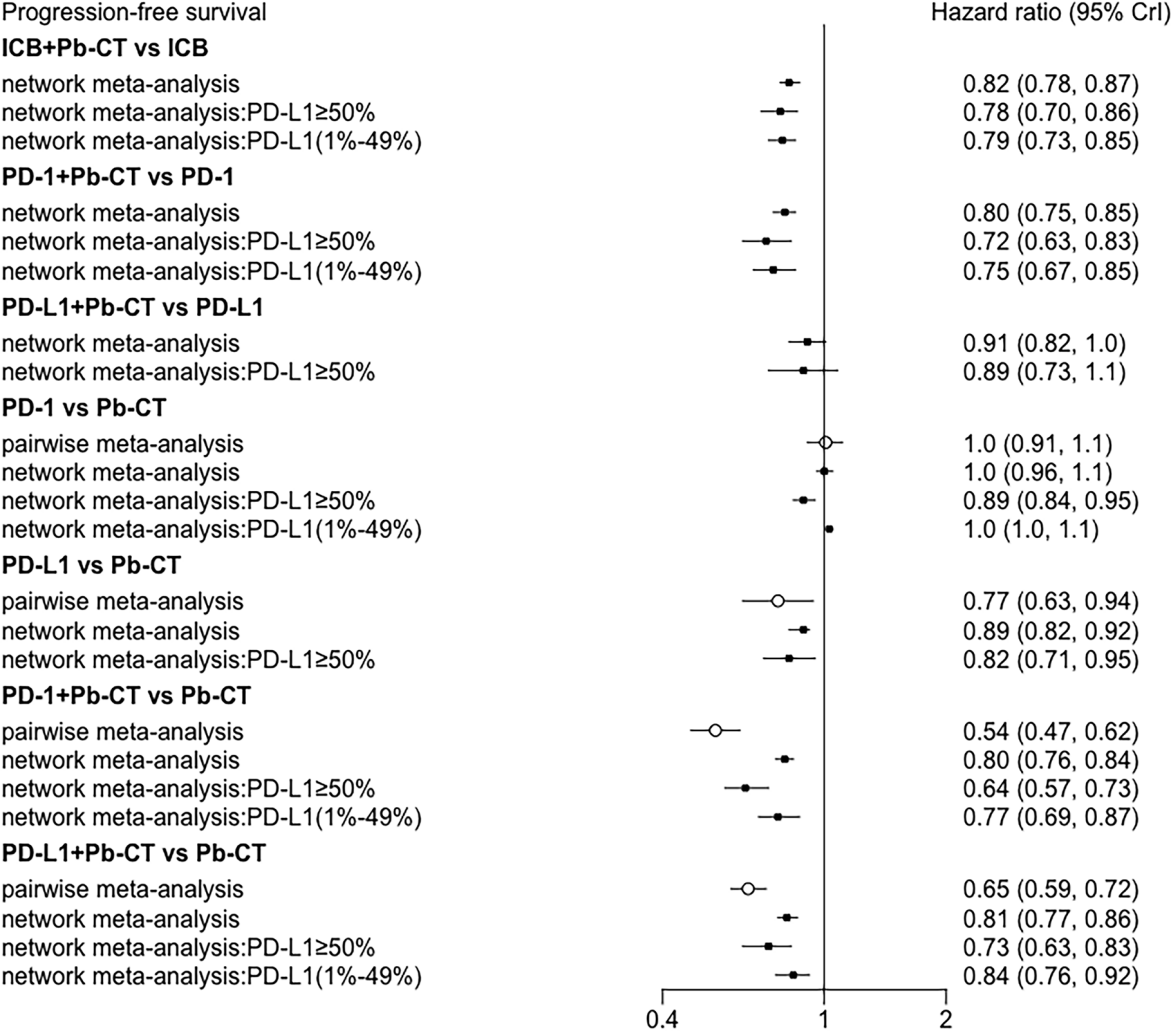
Consistency analysis for progression-free survival.

Node-split plots for ORR and AEs were listed (see **Supplementary Material**) and consistency was confirmed for p-values > 0.05. Forest plots of direct and indirect comparisons were generated for OS (**Figure 8**), PFS (**Figure 9**), ORR (**Supplementary Material**), and AEs (**Supplementary Material**). Funnel plots indicated little report bias among trials (**Supplementary Material**).

## Discussion

Immunotherapy has been proved to be an effective treatment in NSCLC and its use has gradually increased in clinical practice. The appropriate choice of ICI and treatment regime requires solid evidence. ICIs monotherapy and ICIs with chemotherapy are both recommended in NCCN guidelines. ICIs target T-cell regulatory pathways to enhance the anti-tumor immune response, providing significant benefit in cancer therapy (3, 4). Further, ICIs as monotherapy have performed better than chemotherapy alone in several large multicenter trials (9, 10, 13). The NCCN NSCLC panel has recommended single-agent pembrolizumab as first-line treatment for eligible patients with metastatic advanced NSCLC regardless of histology for patients with PD-L1 expression levels greater than 50% and without EGFR, ALK, ROS1, and BRAF V600E mutations. Pembrolizumab is also recommended as monotherapy in patients with low PD-L1 expression (1%–49%) (15), and recent research has suggested that patients with PD-L1 expression levels just below and just above 50% are likely to have a similar response (28). Combining ICIs and chemotherapy sometimes performed better than ICIs as monotherapy, but there is not consistent clinical evidence to support this treatment approach. This study was designed to provide additional guidance on choosing the optimal treatment plan for NSCLC patients with different PD-L1 expression levels.

10 trials evaluating PD-1/PD-L1 inhibitors were included in this analysis. We considered efficacy outcomes (OS, PFS, and ORR) and safety outcomes (AEs) to compare treatment with PD-1/PD-L1 inhibitor plus chemotherapy with PD-1/PD-L1 inhibitors as monotherapy. The trial results were also examined for effect modifiers. Consistency analysis indicated well-behaved data with robust stability.

Our results demonstrated: (i) PD-1/PD-L1 inhibitors plus chemotherapy performed better than PD-1/PD-L1 inhibitors as monotherapy, particularly in patients with low PD-L1 expression levels (1-49%). (ii) PD-1 inhibitors plus chemotherapy improved OS compared with PD-L1 inhibitors plus chemotherapy. (iii) PD-1/PD-L1 inhibitors plus chemotherapy caused more AEs than PD-1/PD-L1 inhibitors as monotherapy.

PD-1/PD-L1 inhibitors plus chemotherapy improved PFS compared with PD-1/PD-L1 inhibitors as monotherapy, but no significant differences were observed for OS. Nonsynonymous mutations and neo-antigens in tumors are associated with improved efficacy, durable clinical benefits, and PFS (5). Stratifying patients based on PD-L1 expression demonstrated that patients with lower PD-L1 expression level (1%–49%) may obtain better OS and PFS benefits from immunotherapy plus chemotherapy compared to immunotherapy alone. Likewise, patients receiving PD-1 inhibitors plus chemotherapy had improved OS and PFS compared with patients receiving PD-1 inhibitors as monotherapy, regardless of PD-L1 expression level. Multiple factors may impact the clinical efficacy of immunotherapy (29). In such cases, ICIs plus chemotherapy may be a better option for patients (30) than platinum-based chemotherapy (11, 23–25, 27).

Basic research has revealed that synergistic anti-tumor effects of immunotherapy and chemotherapy could be mediated through several pathways. First, cytotoxic T lymphocyte (CTL) could generate an immune response to kill tumor cells by releasing perforin or cytokines or through FasL-mediated apoptosis. Chemotherapy increased the expression level of mannose-6-phosphate receptors, with more Granzyme B expressed on CTLs allowing them to enter tumor cells (31). Second, chemotherapy can enhance tumor cell immunogenicity by increasing HSP 70/90 expression on the surface of tumor cells or increasing DNA cross-linking. Tumors with high autophagy would release more adenosine triphosphate than those lacking autophagy, as the latter cannot adequately stimulate T lymphocytes or recruit CD4 and CD8, while the former could raise more dendritic cells (DC) and T lymphocytes (32). Third, chemotherapy causes immunogenic cell death in tumors, causing calreticulin/HSP exposure and adenosine triphosphate/HMGB1 (high-mobility group box 1) release (33). Fourth, chemotherapy could help clear immune-suppressing cells. Along with decreased lymphocytes, immunosuppressive CD4+, CD25+, Foxp3+ regulatory T cells, and myeloid derived suppressor cells could also be cleared (34). Finally, chemotherapy could change the tumor micro-environment to promote antigen presentation and anti-tumor immune response, causing tumor cells to release HMGB1. This could recruit and activate DC as well as induce DC maturation. The combination of HMGB1 and toll-like receptor 4 on DC may also prevent degradation of tumor antigens (35).

PD-L1 expression level may serve as a biomarker to predict ICI efficacy. In this study, patients with lower PD-L1 expression levels (1%–49%) obtained improved survival benefits from PD-1/PD-L1 inhibitors plus chemotherapy compared with PD-1/PD-L1 inhibitors as monotherapy. Meanwhile, PD-1/PD-L1 inhibitors plus chemotherapy showed no survival advantage for patients with high PD-L1 expression level (≥ 50%). Recent research has revealed that T cells secrete cytokines, inducing multiple positive feedback loops. Chemokines promote T cell infiltration, and the altered antigen presentation could help T cells recognize tumor cells. Accordingly, the activation of the PD-1 or PD-L1 pathway is more important than PD-L1 expression level (36).

Interestingly, when PD-1 inhibitors plus chemotherapy were compared with PD-L1 inhibitors plus chemotherapy, the former improved OS, but no significant difference was observed for PFS. Patient stratification according to PD-L1 expression level removed any advantage for OS, PFS, and ORR. Pembrolizumab plus chemotherapy did have a significant advantage compared with atezolizumab plus chemotherapy in terms of OS and PFS.

AEs caused by ICIs may be enhanced by chemotherapy as these treatments cause different kinds of toxicities. Other studies have shown that severe AEs after immune therapy, and especially immune-related AEs, hinder the efficacy of immune treatments. Previous studies have revealed several mechanisms causing AEs (37). Immune therapy increases T-cell activity against antigens present on both tumors and healthy tissue, and immune therapy causes increased levels of preexisting autoantibodies. In addition, immune therapy increases the level of inflammatory cytokines and enhances complement-mediated inflammation due to direct binding of an anti-CTLA-4 antibody to CTLA-4 expressed by normal tissue (38).

Compared with PD-L1 inhibitor monotherapy, PD-1 inhibitor monotherapy improved treatment outcomes in multiple tumor types (39). In treating NSCLC, no significant differences were observed between monotherapy with these two ICIs. PD-1 inhibitors block the interaction between PD-1 and B7.1/PD-L1, while PD-L1 inhibitors block the interaction between PD-L1 and PD-1/RGMB. Once the PD-1-PD-L1 pathway is suppressed, T cells can kill tumor cells. This may explain the similar efficacy of PD-1 inhibitor monotherapy and PD-L1 inhibitor monotherapy in terms of OS, PFS, ORR, and AEs.

## Implication

This network analysis provides new evidence which helps clinician to choose optimal treatments for previously untreated advanced NSCLC patients. PD-1/PD-L1 inhibitors plus chemotherapy did not significantly improve survival compared to PD-1/PD-L1 monotherapy. However, PD-1 inhibitors plus chemotherapy did significantly improve OS, PFS, and ORR, compared to PD-1 inhibitor monotherapy, but also caused increased AEs. PD-L1 inhibitors plus chemotherapy showed no significant improvement in OS, PFS, or ORR compared with PD-L1 inhibitor monotherapy. In addition, patients were stratified according to their PD-L1 expression level. Our results suggest that patients with high PD-L1 expression level (≥ 50%) might be optimally treated with PD-1/PD-L1 inhibitor monotherapy, while patients with low PD-L1 expression level (1-49%) may obtain more benefits from PD-L1 inhibitors plus chemotherapy compared with PD-1 inhibitors as monotherapy, as long as patients could tolerate increased immune-related AEs. Consistent with the comparison between PD-1 inhibitors and PD-L1 inhibitors given as monotherapy, no significant differences in PFS, ORR, or AEs were observed between PD-1 inhibitors plus chemotherapy and PD-L1 inhibitors plus chemotherapy.

## Limitations

This study has several limitations. Due to the essence of network analysis, this research provides a starting point for clinical practice. Several of the multicenter RCTs included in this study are ongoing and this research will need to be updated as more data are available. In addition, patients were not stratified according to histology (adenocarcinoma or squamous cell cancer) for analysis. Another limitation was the high heterogeneity observed when comparing PD-1 to platinum-based chemotherapy, both in OS (I^2^ = 75%) and PFS (I^2^ = 91%). In subgroup analysis, we did not show efficiency in patients with negative PD-L1 expression (<1%), because data within these patients were not available in enrolled published studies, which needs further exploration. Furthermore, this study only included trials evaluating the PD-L1 inhibitor atezolizumab as trials with durvalumab did not meet inclusion criteria. Finally, the KEYNOTE studies measured PD-L1 expression level with 22C3 pharmDx assays and IMpower trials used SP142 assays. These difference in detection methods could potentially cause patients to be misclassified (38).

## Conclusions

PD-1 inhibitors combined with chemotherapy improved outcomes for patients with late-stage NSCLC compared with PD-1 inhibitor monotherapy, while PD-L1 inhibitors combined with chemotherapy had similar outcomes as PD-L1 monotherapy. The survival benefits of PD-1/PD-L1 inhibitors combined with chemotherapy were particularly striking in patients with low PD-L1 expression levels.

## Data Availability Statement

The original contributions presented in the study are included in the article/**Supplementary Material**. Further inquiries can be directed to the corresponding authors.

## Author Contributions

NW, MZ, and XL conceptualized and designed the study, collected and organized the data, and drafted the initial manuscript. XL, SY, YW, and SL collected and organized the data, review the included articles, and conducted the analyses. XL, JY, CL, and JZ collected and organized the data and reviewed the included articles. NW and MZ conceptualized and designed the study, coordinated and supervised data collected, and critically reviewed and revised the manuscript. YY critically reviewed and revised the manuscript. All authors contributed to the article and approved the submitted version.

## Funding

This study was supported by National Key R&D Program of China (No. 2018YFC0910700), National Natural Science Foundation of China (No. 81972842), the Capital Health Research and Development of Special Funds (No. 2020-2-2154), Beijing Natural Science Foundation (No. 7192036), Beijing Municipal Administration of Hospitals’Youth Programme (No. QML20191107).

## Conflict of Interest

The authors declare that the research was conducted in the absence of any commercial or financial relationships that could be construed as a potential conflict of interest.
