# Supplementary material for: Efficacy and Safety of PD-1/PD-L1 Inhibitors Plus Chemotherapy Versus PD-1/PD-L1 Inhibitors in Advanced Non-Small Cell Lung Cancer: A Network Analysis of Randomized Controlled Trials
Source: Front Oncol. 2021 Jan 11;10:574752. doi: 10.3389/fonc.2020.574752 (PMC7873939; doi:10.3389/fonc.2020.574752)
Supplement: Supplementary file 1 [file DataSheet_1.pdf]

PRISMA NMA Checklist of Items to Include When Reporting A Systematic Review Involving a Network Meta-analysis

Section/Topic	Item #	Checklist Item	Reported on Page #
**TITLE**			**1**
Title	1	Identify the report as a systematic review *incorporating a network meta-analysis (or related form of meta-analysis).*	**1**
**ABSTRACT**			**1-2**
Structured summary	2	Provide a structured summary including, as applicable:<br>**Background:** main objectives<br>**Methods:** data sources; study eligibility criteria, participants, and interventions; study appraisal; and *synthesis methods, such as network meta-analysis.*<br>**Results:** number of studies and participants identified; summary estimates with corresponding confidence/credible intervals; *treatment rankings may also be discussed. Authors may choose to summarize pairwise comparisons against a chosen treatment included in their analyses for brevity.*<br>**Discussion/Conclusions:** limitations; conclusions and implications of findings.<br>**Other:** primary source of funding; systematic review registration number with registry name.	
**INTRODUCTION**			
Rationale	3	Describe the rationale for the review in the context of what is already known*, including mention of why a network meta-analysis has been conducted.*	2
Objectives	4	Provide an explicit statement of questions being addressed, with reference to participants, interventions, comparisons, outcomes, and study design (PICOS).	2
**METHODS**			
Protocol and registration	5	Indicate whether a review protocol exists and if and where it can be accessed (e.g., Web address); and, if available, provide registration information, including registration number.	4
Eligibility criteria	6	Specify study characteristics (e.g., PICOS, length of follow-up) and report characteristics (e.g., years considered, language, publication status) used as criteria for eligibility, giving rationale. *Clearly*	4

		describe eligible treatments included in the treatment network, and note whether any have been clustered or merged into the same node (with justification).	
Information sources	7	Describe all information sources (e.g., databases with dates of coverage, contact with study authors to identify additional studies) in the search and date last searched.	4
Search	8	Present full electronic search strategy for at least one database, including any limits used, such that it could be repeated.	4
Study selection	9	State the process for selecting studies (i.e., screening, eligibility, included in systematic review, and, if applicable, included in the meta-analysis).	4
Data collection process	10	Describe method of data extraction from reports (e.g., piloted forms, independently, in duplicate) and any processes for obtaining and confirming data from investigators.	4
Data items	11	List and define all variables for which data were sought (e.g., PICOS, funding sources) and any assumptions and simplifications made.	4
**Geometry of the network**	**S1**	Describe methods used to explore the geometry of the treatment network under study and potential biases related to it. This should include how the evidence base has been graphically summarized for presentation, and what characteristics were compiled and used to describe the evidence base to readers.	7
Risk of bias within individual studies	12	Describe methods used for assessing risk of bias of individual studies (including specification of whether this was done at the study or outcome level), and how this information is to be used in any data synthesis.	5
Summary measures	13	State the principal summary measures (e.g., risk ratio, difference in means). *Also describe the use of additional summary measures assessed, such as treatment rankings and surface under the cumulative ranking curve (SUCRA) values, as well as modified approaches used to present summary findings from meta-analyses.*	5
Planned methods of analysis	14	Describe the methods of handling data and combining results of studies for each network meta-analysis. This should include, but not be limited to:<br>• *Handling of multi-arm trials;*<br>• *Selection of variance structure;*<br>• *Selection of prior distributions in Bayesian analyses; and*<br>• *Assessment of model fit.*	5
**Assessment of Inconsistency**	**S2**	Describe the statistical methods used to evaluate the agreement of direct and indirect evidence in the treatment network(s) studied. Describe efforts taken	6

		to address its presence when found.	
Risk of bias across studies	15	Specify any assessment of risk of bias that may affect the cumulative evidence (e.g., publication bias, selective reporting within studies).	7,10
Additional analyses	16	Describe methods of additional analyses if done, indicating which were pre-specified. This may include, but not be limited to, the following:<br>• Sensitivity or subgroup analyses;<br>• Meta-regression analyses;<br>• *Alternative formulations of the treatment network; and*<br>• *Use of alternative prior distributions for Bayesian analyses (if applicable).*	10
**RESULTS†**			
Study selection	17	Give numbers of studies screened, assessed for eligibility, and included in the review, with reasons for exclusions at each stage, ideally with a flow diagram.	7
**Presentation of network structure**	**S3**	Provide a network graph of the included studies to enable visualization of the geometry of the treatment network.	
**Summary of network geometry**	**S4**	Provide a brief overview of characteristics of the treatment network. This may include commentary on the abundance of trials and randomized patients for the different interventions and pairwise comparisons in the network, gaps of evidence in the treatment network, and potential biases reflected by the network structure.	
Study characteristics	18	For each study, present characteristics for which data were extracted (e.g., study size, PICOS, follow-up period) and provide the citations.	6
Risk of bias within studies	19	Present data on risk of bias of each study and, if available, any outcome level assessment.	
Results of individual studies	20	For all outcomes considered (benefits or harms), present, for each study: 1) simple summary data for each intervention group, and 2) effect estimates and confidence intervals. *Modified approaches may be needed to deal with information from larger networks.*	8
Synthesis of results	21	Present results of each meta-analysis done, including confidence/credible intervals. *In larger networks,*	8-10

		authors may focus on comparisons versus a particular comparator (e.g. placebo or standard care), with full findings presented in an appendix. League tables and forest plots may be considered to summarize pairwise comparisons. If additional summary measures were explored (such as treatment rankings), these should also be presented.	
**Exploration for inconsistency**	**S5**	Describe results from investigations of inconsistency. This may include such information as measures of model fit to compare consistency and inconsistency models, *P* values from statistical tests, or summary of inconsistency estimates from different parts of the treatment network.	
Risk of bias across studies	22	Present results of any assessment of risk of bias across studies for the evidence base being studied.	10
Results of additional analyses	23	Give results of additional analyses, if done (e.g., sensitivity or subgroup analyses, meta-regression analyses*, alternative network geometries studied, alternative choice of prior distributions for Bayesian analyses,* and so forth).	
**DISCUSSION**			
Summary of evidence	24	Summarize the main findings, including the strength of evidence for each main outcome; consider their relevance to key groups (e.g., healthcare providers, users, and policy-makers).	13-14
Limitations	25	Discuss limitations at study and outcome level (e.g., risk of bias), and at review level (e.g., incomplete retrieval of identified research, reporting bias). *Comment on the validity of the assumptions, such as transitivity and consistency. Comment on any concerns regarding network geometry (e.g., avoidance of certain comparisons).*	16
Conclusions	26	Provide a general interpretation of the results in the context of other evidence, and implications for future research.	16
**FUNDING**			
Funding	27	Describe sources of funding for the systematic review and other support (e.g., supply of data); role of funders for the systematic review. This should also include information regarding whether funding has been received from manufacturers of treatments in the network and/or whether some of the authors are content experts with professional conflicts of interest that could affect use of treatments in the network.	16-17

Supplementary Table 1. Checklist of the PRISMA extension for network meta-analysis.
PICOS = population, intervention, comparators, outcomes, study design.

* Text in italics indicateS wording specific to reporting of network meta-analyses that has been added to guidance from the PRISMA statement.

† Authors may wish to plan for use of appendices to present all relevant information in full detail for items in this section.

SEARCHING STRATEGIE

Search **((((((((((((((((((Lung Neoplasms) OR Pulmonary Neoplasms) OR Neoplasms, Lung) OR Lung Neoplasm) OR Neoplasm, Lung) OR Neoplasms, Pulmonary) OR Neoplasm, Pulmonary) OR Pulmonary Neoplasm) OR Lung Cancer) OR Cancer, Lung) OR Cancers, Lung) OR Lung Cancers) OR Pulmonary Cancer) OR Cancer, Pulmonary) OR Cancers, Pulmonary) OR Pulmonary Cancers) OR Cancer of the Lung) OR Cancer of Lung)) AND ((((Chemotherapy, Adjuvant) OR Adjuvant Chemotherapy) OR Drug Therapy, Adjuvant) OR Adjuvant Drug Therapy)) AND ((((((Durvalumab) OR Atezolizumab) OR Nivolumab) OR Pembrolizumab) OR Immune checkpoint blockade) OR Immune checkpoint inhibitor)**

- **The searching strategy in PubMed**

#1 MeSH descriptor: [Lung Neoplasms] explode all trees 7460

#2 MeSH descriptor: [Carcinoma, Non-Small-Cell Lung] explode all trees 4156

#3 (lung):ti,ab,kw (Word variations have been searched) 70013

#4 (pulmon*):ti,ab,kw (Word variations have been searched) 50901

#5 (neoplas*):ti,ab,kw (Word variations have been searched) 80112

#6 (cancer):ti,ab,kw (Word variations have been searched) 159289

#7 (carcinoma*):ti,ab,kw (Word variations have been searched) 41358

#8 #3 or #4 96743

#9 #5 or #6 or #7 189454

#10 #8 AND #9 27830

#11 #10 or #1 or #2 27933

#12 (pembrolizumab):ti,ab,kw (Word variations have been searched) 1281

#13 (Nivolumab):ti,ab,kw (Word variations have been searched) 1525

#14 (Atezolizumab):ti,ab,kw (Word variations have been searched) 608

#15 (Durvalumab):ti,ab,kw (Word variations have been searched) 432

#16 #12 or #13 or #14 or #15 3467

#17 MeSH descriptor: [Drug Therapy] explode all trees 137717

#18 (chemotherap*):ti,ab,kw (Word variations have been searched) 75363

#19 #17 or #18 194330

#20 #11 and #16 and #19 677

- **The searching strategy in Cochrane Library**

#30	#27 AND #28 AND #29	639
#29	#23 OR #24 OR #25 OR #26	27,649
#28	#19 OR #20 OR #21 OR #22	203,476
#27	#1 OR #2 OR #3 OR #4 OR #5 OR #6 OR #7 OR #8 OR #9 OR #10 OR #11 OR #12 OR #13 OR #14 OR #15 OR #16 OR #17 OR #18	595,968
#26	durvalumab	3,654
#25	atezolizumab	5,408
#24	nivolumab	17,848
#23	pembrolizumab	15,487
#22	adjuvant AND drug AND therapy	159,389
#21	drug AND therapy, AND adjuvant	159,389
#20	adjuvant AND chemotherapy	126,966
#19	chemotherapy, AND adjuvant	126,966
#18	cancer AND of AND lung	539,418
#17	cancer AND of AND the AND lung	511,168
#16	pulmonary AND cancers	8,740
#15	cancers AND pulmonary	8,740
#14	cancer, AND pulmonary	114,321
#13	pulmonary AND cancer	114,321
#12	lung AND cancers	70,018
#11	cancers, AND lung	70,018
#10	cancer, AND lung	547,058
#9	lung AND cancer	547,058
#8	pulmonary AND neoplasm	25,442
#7	neoplasm, AND pulmonary	25,442
#6	neoplasms, AND pulmonary	8,618
#5	neoplasms, AND lung	26,753
#4	lung AND neoplasm	109,250
#3	neoplasm, AND lung	109,250
#2	pulmonary AND neoplasm	25,442
#1	lung AND neoplasms	26,753

- **The searching strategy in Embase**

Studies	Years	Trials	Excluding reasons
Marina Chiara Garassino	2018	ATLANTIC	Patients may have EGFR/ALK mutation
M. Reck	2013	-	Anti-CTLA-4 was involved
Ramaswamy Govindan	2017	-	Anti-CTLA-4 was involved
Matthew D Hellmann	2017	CheckMate-012	Anti-CTLA-4 was involved
Martin Reck	2016	-	Anti-CTLA-4 was involved; small-cell lung cancer
S.J. Antonia	2017	PACIFIC	Radiotherapy was involved
M.A. Socinski	2018	IMpower-150	Anti-CTLA-4 was involved
Scott J Antonia	2016	CheckMate-032	Anti-CTLA-4 was involved
Roy S Herbst	2016	KEYNOTE-010	Second-line treatment with docetaxel
Julie Brahmer	2015	CheckMate-017	Second-line treatment with docetaxel
Hossein Borghaei	2015	CheckMate-057	Second-line treatment with docetaxel
Louis Fehrenbacher	2016	POPLAR	Second-line treatment with docetaxel
Achim Rittmeyer	2017	OAK	Second-line treatment with docetaxel
Shuai Wang	2018	-	Without necessary endpoints

Supplementary Table 2. Reasons of excluding trials

1, Survival：Pooled estimates of subgroup analysis (OS and PFS).

Overall survival

Progression-free survival

**PD-1/PD-L1**	0.94 (0.90 to 1.01)	1.08 (1.03 to 1.12)
1.21 (1.15 to 1.28)	**PD-1/PD-L1+Pb-CT**	1.14 (1.09 to 1.19)
0.98 (0.94 to 1.02)	0.81 (0.78 to 0.84)	**Pb-CT**

Supplementary Figure 1. Immune checkpoint blockade related survival

(Note: Pb-CT=platinum-based chemotherapy)

Overall survival

Progression-free survival

**PD-1/PD-L1**	0.94 (0.82 to 1.07)	1.17 (1.1 to 1.25)
1.21 (1.16 to 1.44)	**PD-1/PD-L1+Pb-CT**	1.14 (1.12 to 1.41)
0.98 (0.83 to 0.93)	0.68 (0.62 to 0.74)	**Pb-CT**

Supplementary Figure 2. Immune checkpoint blockade related survival (PD-L1>=50%)

(Note: ICB=anti-PD-1/PD-L1; Pb-CT=platinum-based chemotherapy)

Overall survival

Progression-free survival

**PD-1/PD-L1**	0.84 (0.73 to 0.96)	1.04 (0.96 to 1.12)
1.27 (1.18 to 1.37)	**PD-1/PD-L1+Pb-CT**	1.24 (1.11 to 1.39)
1.03 (1.01 to 1.05)	0.81 (0.76 to 0.87)	**Pb-CT**

Supplementary Figure 3. Immune checkpoint blockade related survival (PD-L1:1%-49%)

(Note: ICB=anti-PD-1/PD-L1; Pb-CT=platinum-based chemotherapy)

Overall survival

Progression-free survival

PD-1	0.99 (0.88 to 1.11)	0.84 (0.77 to 0.92)	0.99 (0.92 to 1.07)	1.07 (1.02 to 1.13)
1.12 (1.02 to 1.24)	PD-L1	0.85 (0.74 to 0.97)	1.01 (0.89 to 1.13)	1.08 (0.97 to 1.21)
1.25 (1.17 to 1.33)	1.11 (1.01 to 1.23)	PD-1+Pb-CT	1.18 (1.07 to 1.31)	1.28 (1.18 to 1.38)
1.23 (1.15 to 1.32)	1.10 (0.99 to 1.22)	0.99 (0.92 to 1.06)	PD-L1+Pb-CT	1.08 (1.03 to 1.14)
1.002 (0.96 to 1.05)	0.89 (0.82 to 0.97)	0.80 (0.76 to 0.84)	0.81 (0.77 to 0.86)	Pb-CT

Supplementary Figure 4. PD-(L)1- related survival of all patients

(Note: Pb-CT=platinum-based chemotherapy)

Overall survival

Progression-free survival

**PD-1**	0.92 (0.76 to 1.11)	0.86 (0.73,1.03)	0.995 (0.83,1.19)	1.16 (1.08,1.25)
1.09 (0.93 to 1.27)	**PD-L1**	0.94 (0.74,1.18)	1.08 (0.85,1.37)	1.26 (1.06,1.44)
1.38 (1.20 to 1.58)	1.27 (0.78 to 1.53)	**PD-1+Pb-CT**	1.15 (0.92,1.44)	1.34 (1.15,1.57)
1.23 (1.05 to 1.42)	1.13 (0.92 to 1.37)	0.89 (0.74 to 1.07)	**PD-L1+Pb-CT**	1.17 (0.99,1.37)
0.89 (0.84 to 0.95)	0.82 (0.71 to 0.95)	0.64 (0.57 to 0.73)	0.73 (0.63 to 0.83)	**Pb-CT**

Supplementary Figure 5. PD-(L)1-related survival（PD-L1>=50%）

(Note: Pb-CT=platinum-based chemotherapy)

Overall survival

Progression-free survival

**PD-1**	0.81 (0.68 to 0.95)	0.89 (0.72 to 1.09)	1.04 (0.96 to 1.12)
1.33 (1.18 to 1.50)	**PD-1+Pb-CT**	1.10 (0.87 to 1.40)	1.29 (1.11 to 1.49)
1.23 (1.12 to 1.36)	0.93 (0.80 to 1.07)	**PD-L1+Pb-CT**	1.17 (0.96 to 1.41)
1.03 (1.01 to 1.05)	0.77 (0.69 to 0.87)	0.84 (0.76 to 0.92)	**Pb-CT**

Supplementary Figure 6. PD-(L)1 related survival (PD-L1(1%-49%))

(Note: Pb-CT=platinum-based chemotherapy)

Overall survival (upper triangle); Progression-free survival (lower triangle)

**Pembrolizumab**	1.14 (1.03 to 1.28)	1.03 (0.91 to 1.16)	0.87 (0.79 to 0.96)	1.03 (0.95 to 1.11)	1.11 (1.05 to 1.18)
0.92 (0.83 to 1.02)	**Nivolumab**	0.90 (0.78 to 1.03)	0.76 (0.67 to 0.86)	0.90 (0.81 to 0.999)	0.99 (0.88 to 1.07)
1.10 (0.99 to 1.21)	1.19 (1.04 to 1.36)	**Atezolizumab**	0.85 (0.74 to 0.97)	1.002 (0.89 to 1.13)	1.08 (0.97 to 1.21)
1.29 (1.19 to 1.39)	1.40 (1.26 to 1.56)	1.17 (1.05 to 1.3)	**Pembrolizumab+Pb-CT**	1.18 (1.07 to 1.30)	1.28 (1.18 to 1.38)
1.18 (1.10 to 1.27)	1.29 (1.17 to 1.43)	1,08 (0.98 to 1.19)	0.92 (0.85 to 0.99)	**Atezolizumab+Pb-CT**	1.08 (1.03 to 1.14)
0.98 (0.98 to 1.03)	1.07 (0.98 to 1.17)	0.89 (0.82 to 0.97)	0.76 (0.72 to 0.87)	0.83 (0.79 to 0.87)	**Pb-CT**

Supplementary Figure 7. Survival of different ICIs of all patients

(Note: Pb-CT=platinum-based chemotherapy)

2, Pairwise meta-analysis forest plots for survival (OS and PFS)

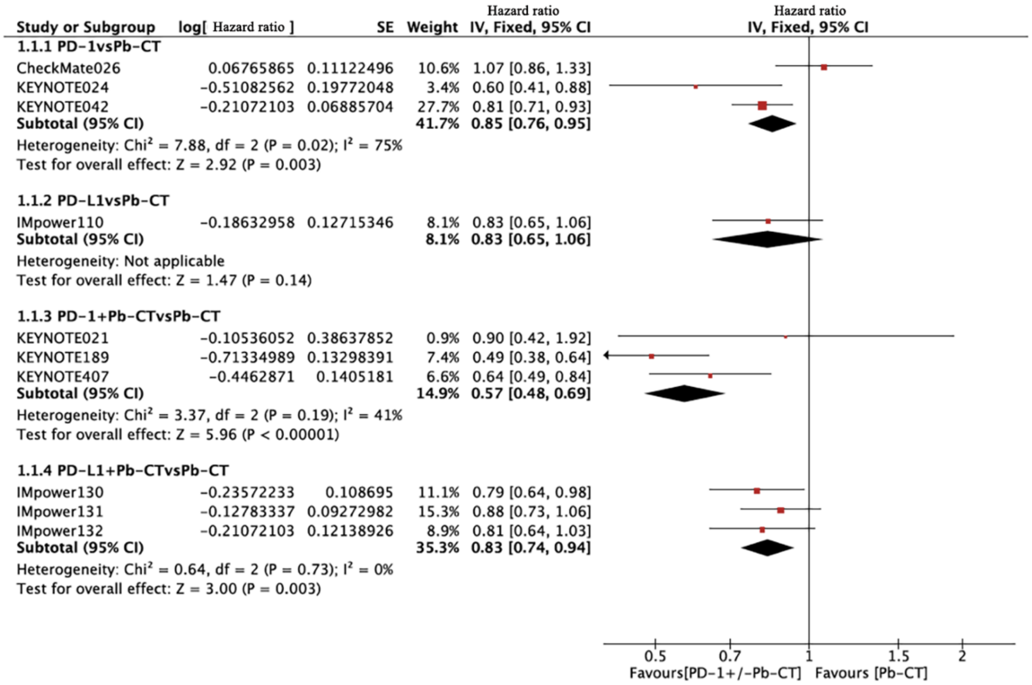

Supplementary Figure 8. Pairwise meta-analysis for overall survival

(Note: Pb-CT=platinum-based chemotherapy)

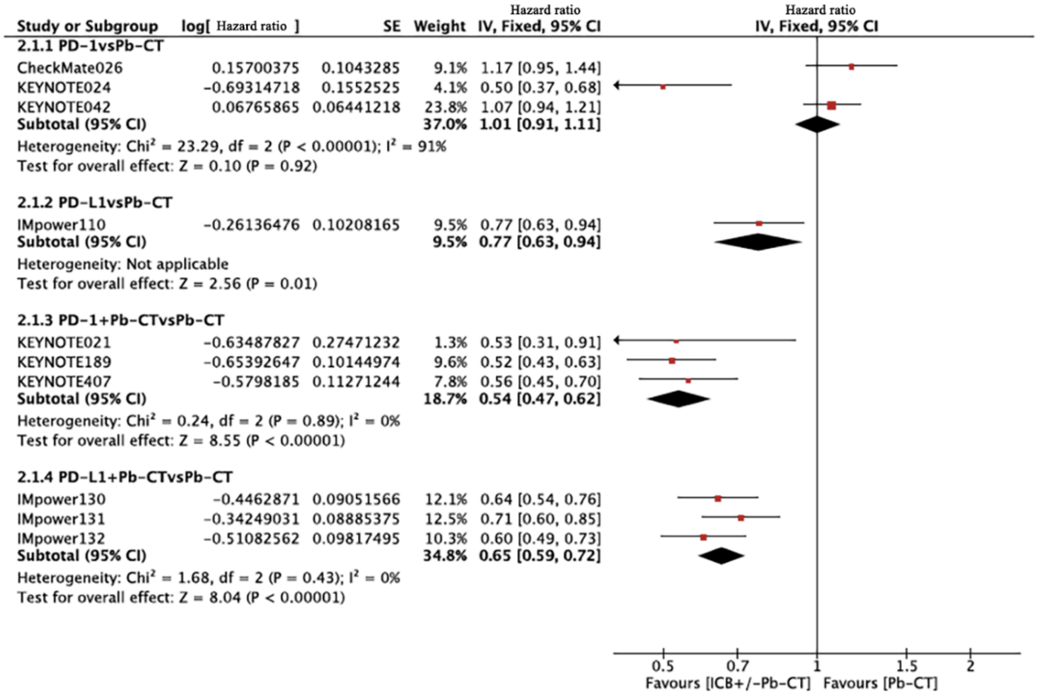

Supplementary Figure 9. Pairwise meta-analysis for progression-free survival

(Note: ICB=anti-PD-1/PD-L1; Pb-CT=platinum-based chemotherapy)

3, Consistency and inconsistency analysis for survival

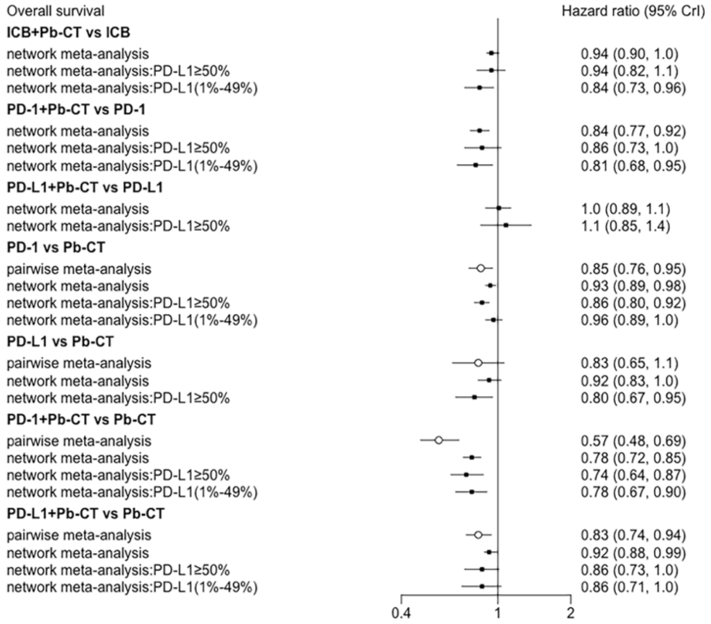

Supplementary Figure 10. Consistency analysis for overall survival

(Note: ICB=anti-PD-1/PD-L1; Pb-CT=platinum-based chemotherapy)

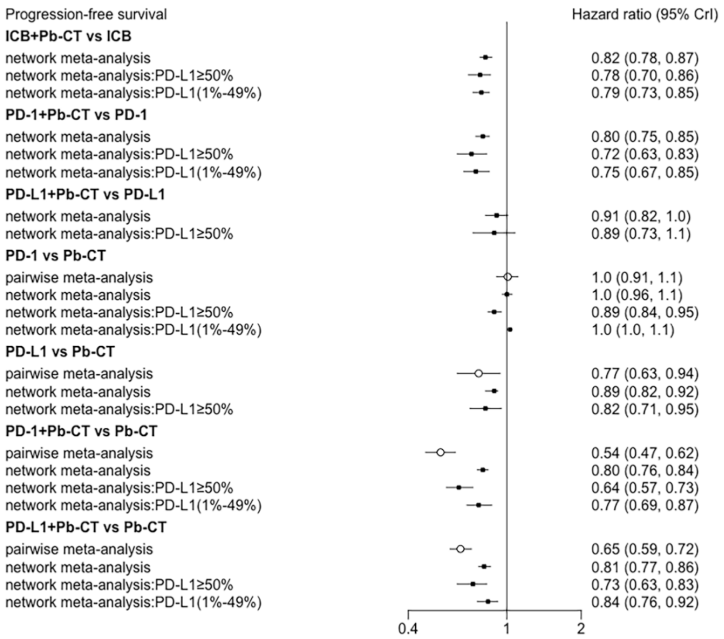

Supplementary Figure 11. Consistency analysis for progression-free survival

(Note: ICB=anti-PD-1/PD-L1; Pb-CT=platinum-based chemotherapy)

4, Ranking probability for survival

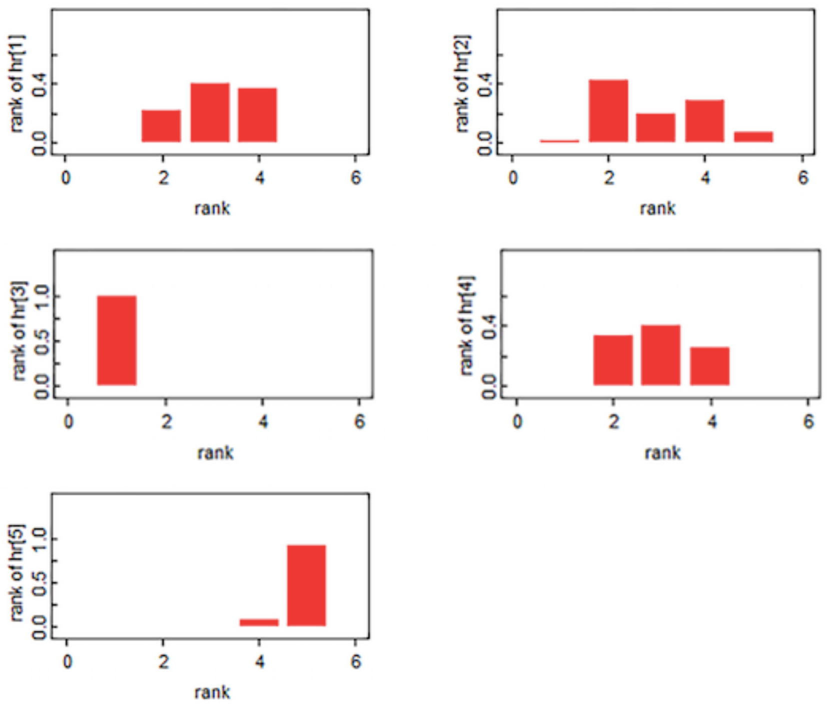

Supplementary Figure 12. Rank of overall survival

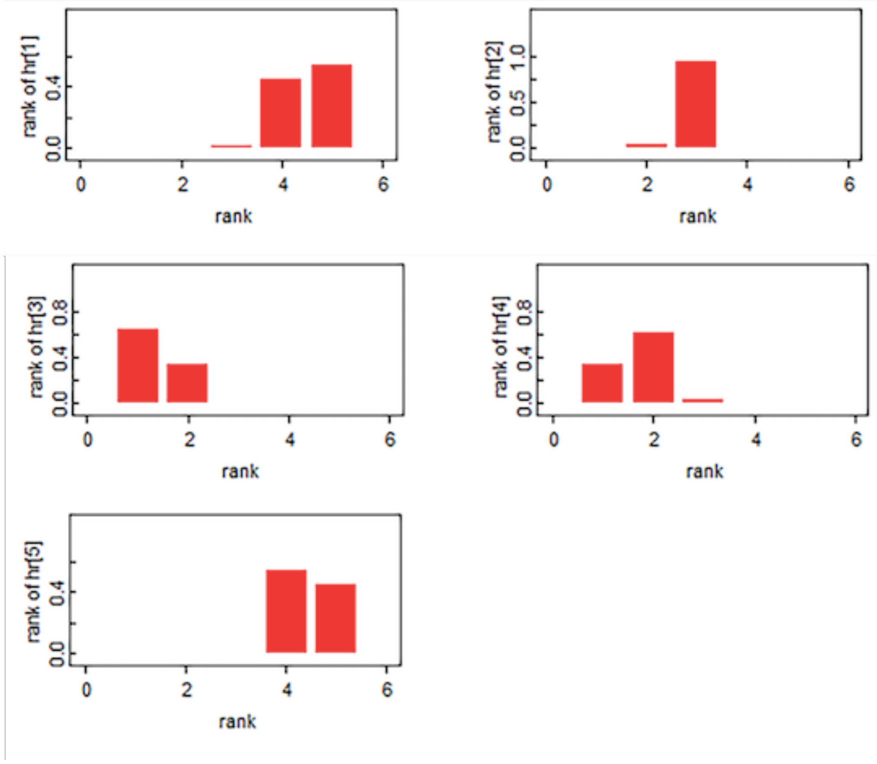

Supplementary Figure 13. Rank of progression-free survival

Treatments	Rank of possibilities (%)					
	1	2	3	4	5	6
Overall survival for advance NSCLC						
1: PD-1	0	0	99	0	0	-
2: PD-L1	22.1	43.1	0	33.9	0	-
3: PD-1+Pb-CT	40.4	19.4	0	40.2	0	-
4: PD-L1+Pb-CT	37.4	29.5	0	26	0	-
5: Pb-CT	0	0	0	0	90	-
Progression-free survival for advanced NSCLC						
1: PD-1	0	0	64.6	34.6	0	-
2: PD-L1	0	0	34	61.7	0	-
3: PD-1+Pb-CT	0	94	0	0	0	-
4: PD-L1+Pb-CT	45	0	0	0	54	-
5: Pb-CT	54	0	0	0	46	-
Overall survival for advanced NSCLC						
1: Pembrolizumab	2	52.5	33.5	12.9	1	0
2: Nivolumab	0	1.5	3.4	10.3	29.8	55.1
3: Atezolizumab	0	29.5	23.3	32.4	9.9	4.1
4: Pembrolizumab+Pb-CT	98.9	1	0	0	0	0
5: Atezolizumab+Pb-CT	0	15.6	39.8	40.1	4.4	0
6: Pb-CT	0	0	0	4.24	55	40.7
Progression-free survival for advanced NSCLC						
1: Pembrolizumab	0	0	3	70	22.2	4.5
2: Nivolumab	0	0	0	6.5	7.1	86.1
3: Atezolizumab	0	7.2	89	3.1	0	0
4: Pembrolizumab+Pb-CT	98.4	1.6	0	0	0	0
5: Atezolizumab+Pb-CT	1.5	91.2	7.4	0	0	0
6: Pb-CT	0	0	0	20.3	70.2	9.4

Supplementary Table 3. Ranks of ICIs in survival

5, Iteration history for survival

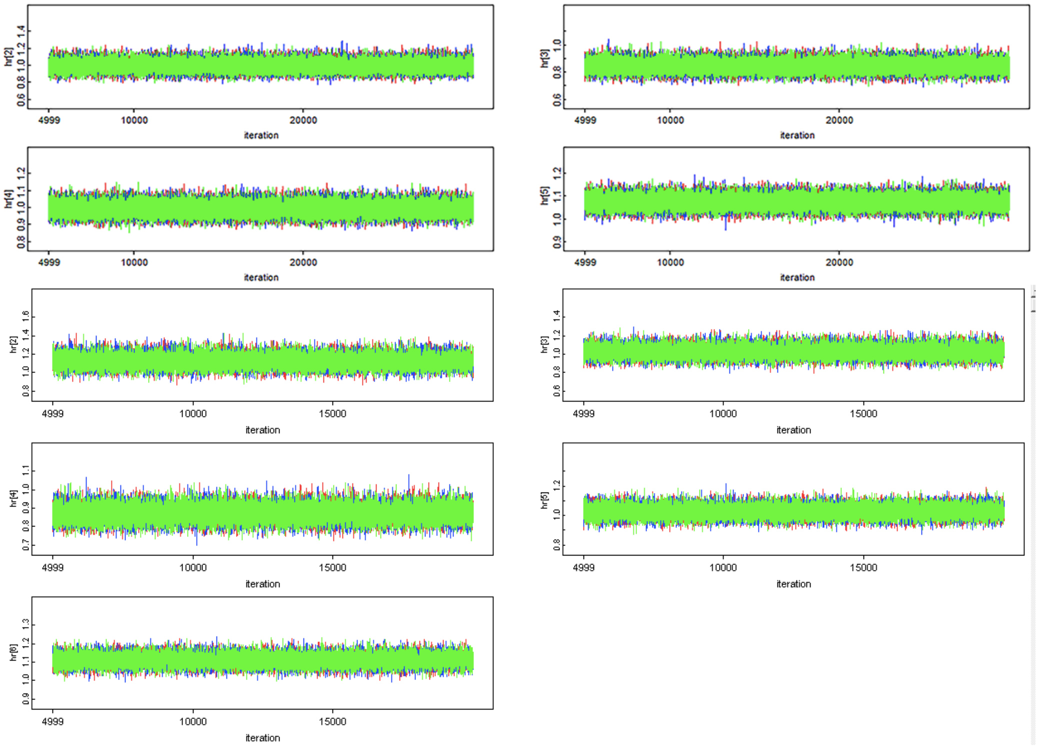

Supplementary Figure 14. Iteration history for overall survival

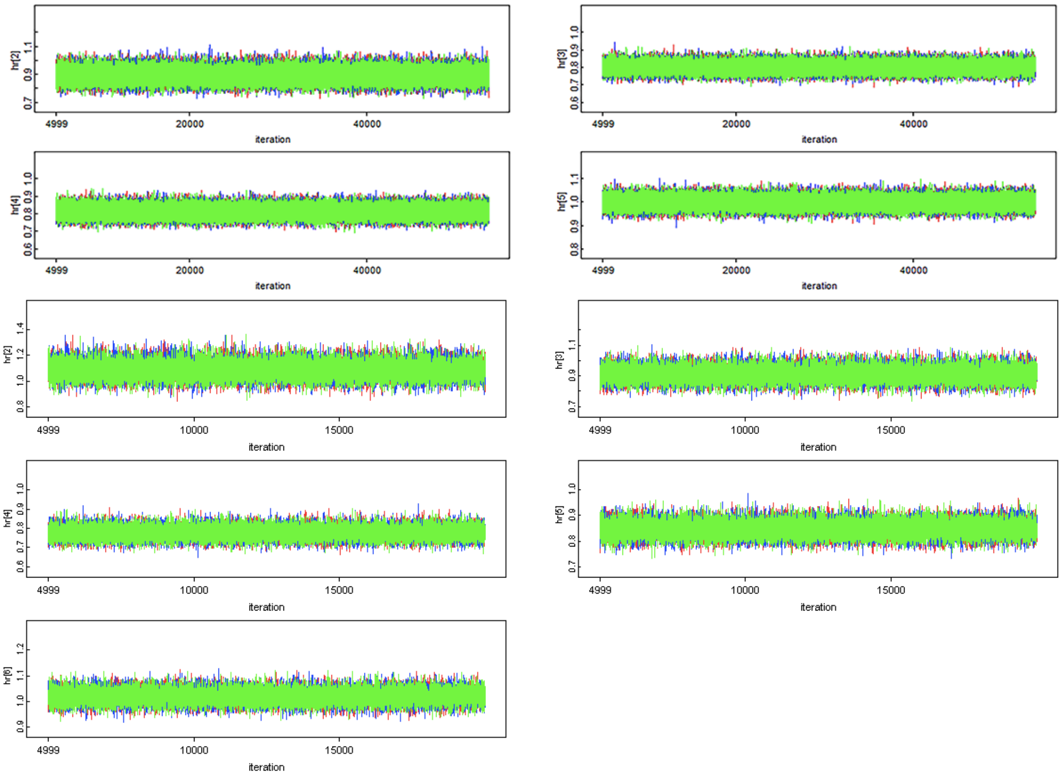

Supplementary Figure 15. Iteration history for progression-free survival

6, Quantiles plots of survival

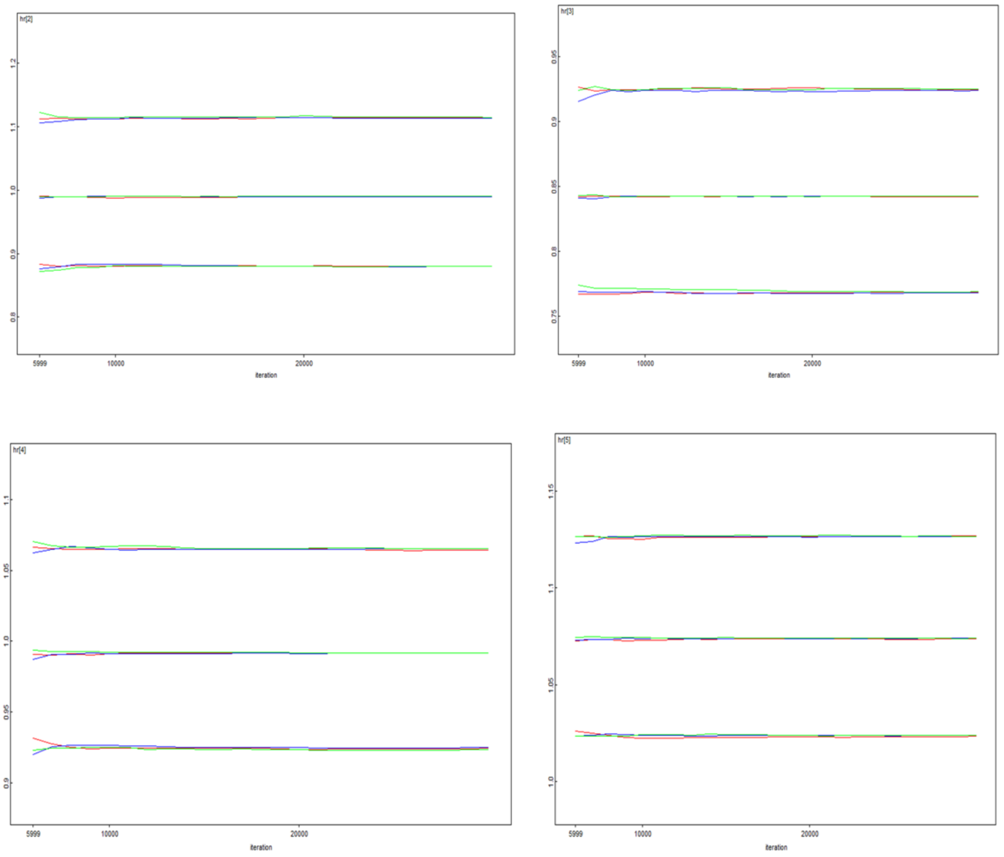

Supplementary Figure 16. Quantile plot of overall survival

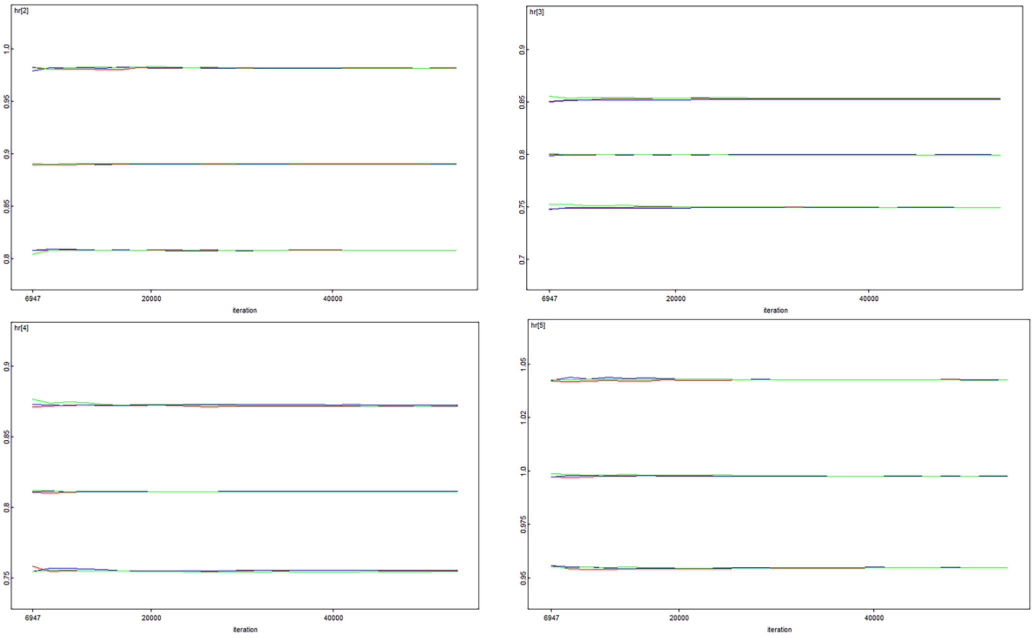

Supplementary Figure 17. Quantile plot of Progression-free survival

6, Density plots of survival

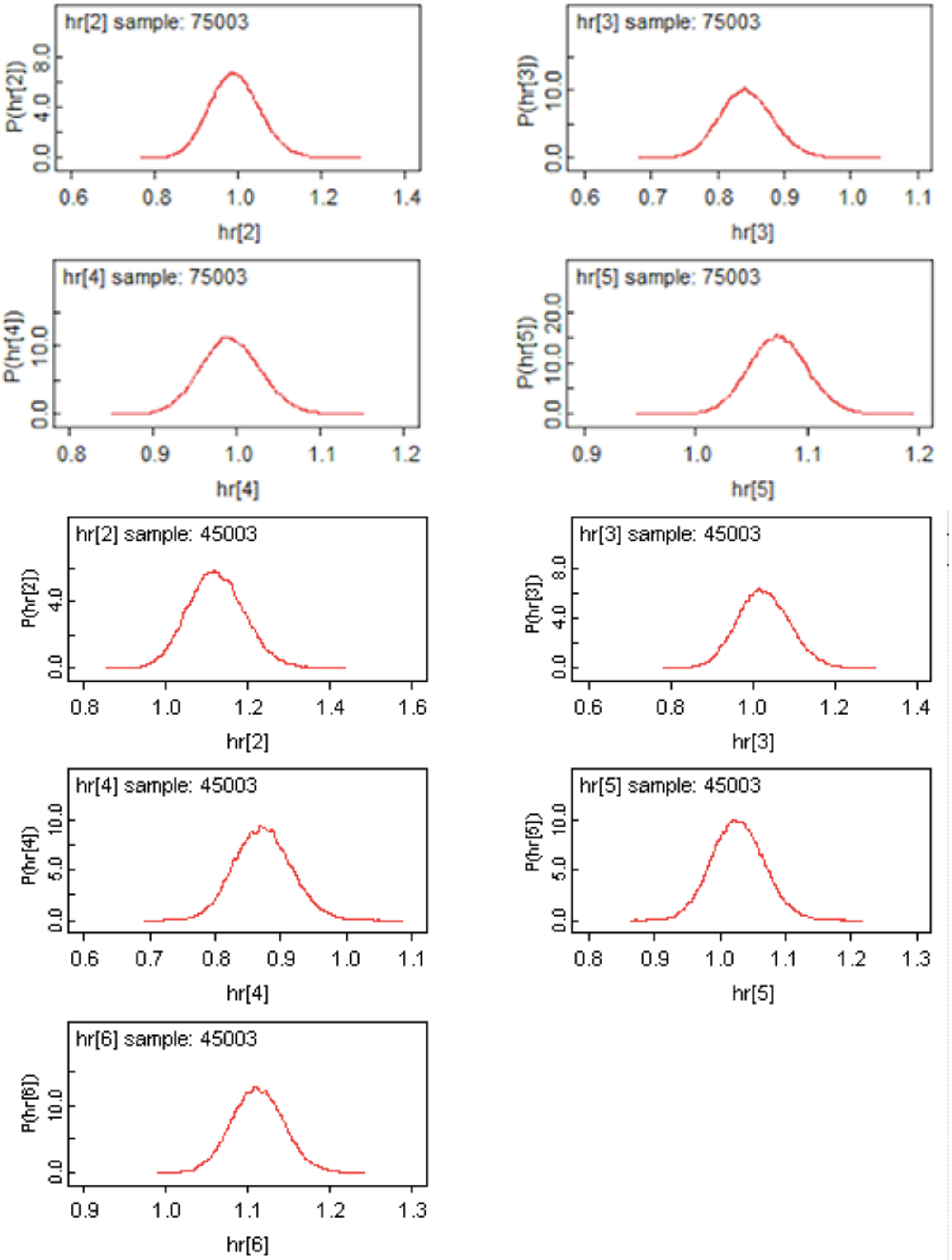

Supplementary Figure 18. Density plots of overall survival

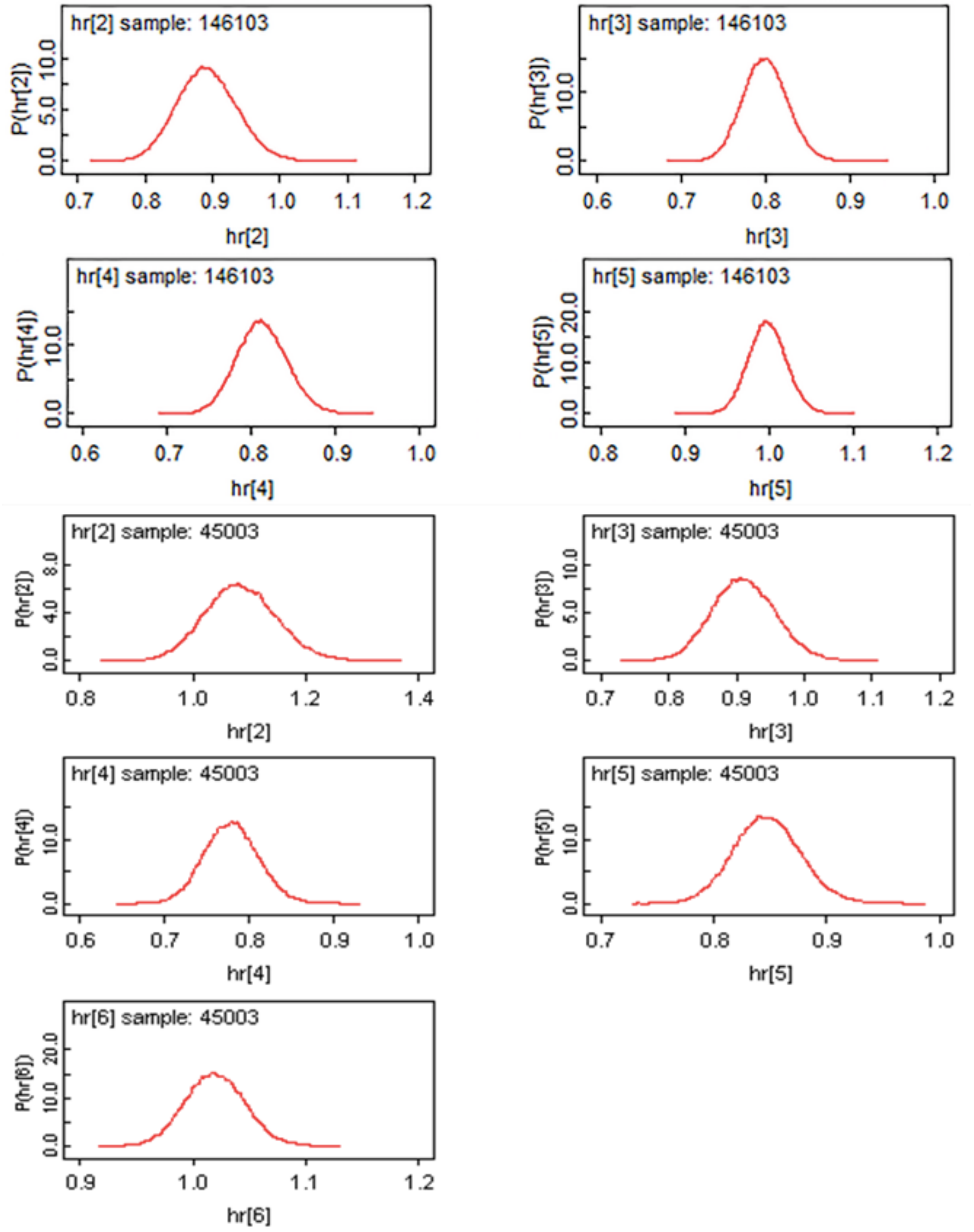

Supplementary Figure 19. Density plots of Progression-free survival

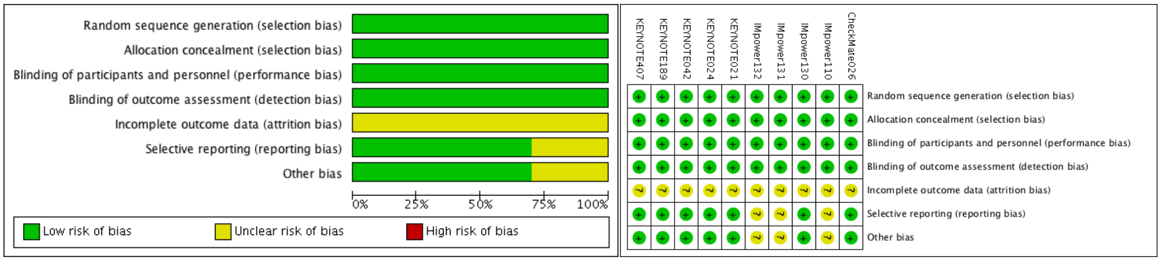

Supplementary Figure 20. Bias of included trials

7, Pooled estimates Objective response rate (ORR)

**PD-1**	0.80 (0.28 to 2.31)	2.55 (1.20 to 5.28)	1.62 (0.75 to 3.51)	0.90 (0.51 to 1.55)
1.25 (0.43 to 3.56)	**PD-L1**	3.18 (1.08 to 8.80)	2.02 (0.68 to 5.80)	1.12 (0.43 to 2.80)
0.39 (0.19 to 0.84)	0.31 (0.11 to 0.93)	**PD-1+Pb-CT**	0.63 (0.31 to 1.36)	0.35 (0.22 to 0.59)
0.62 (0.29 to 1.34)	0.50 (0.17 to 1.48)	1.58 (0.73 to 3.21)	**PD-L1+Pb-CT**	0.56 (0.33 to 0.96)
1.11 (0.65 to 1.95)	0.89 (0.36 to 2.31)	2.82 (1.71 to 4.58)	1.79 (1.05 to 3.07)	**Pb-CT**

Supplementary Figure 21. Objective response rate (OR 95% confidence interval)

(Note: Pb-CT=platinum-based chemotherapy)

8, Ranks of Objective response rate

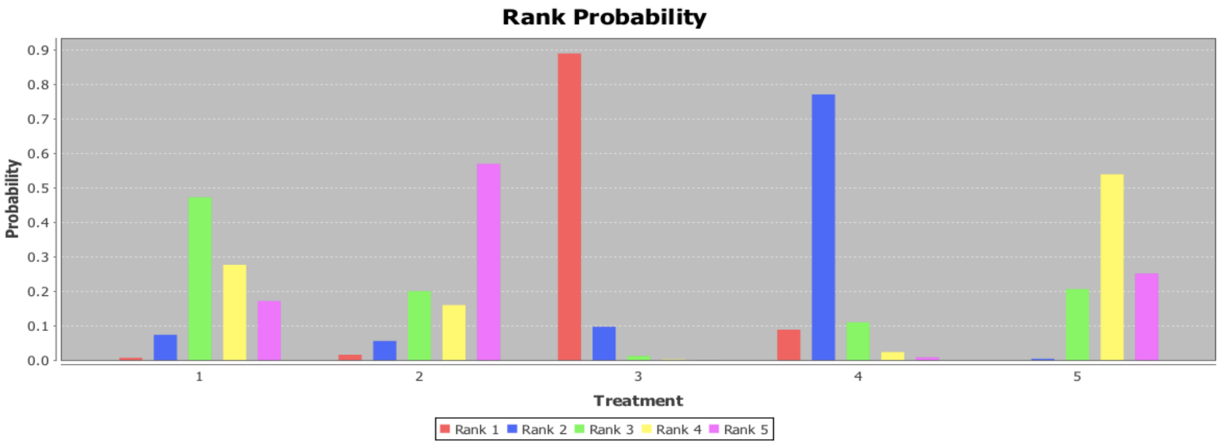

Supplementary Figure 22. Ranking plot of objective response rate

(**Note**: **1-5**: **PD-1, PD-L1, PD-1+Pb-CT, PD-L1+Pb-CT, Pb-CT; Pb-CT=platinum-based chemotherapy)**

Treatments	Rank of possibilities (%)				
	1	2	3	4	5
Objective response rate of advanced NSCLC					
1: PD-1	1	7	47	28	17
2: PD-L1	2	6	20	16	57
3: PD-1+Pb-CT	89	10	1	0	0
4: PD-L1+Pb-CT	9	77	11	2	1
5: Pb-CT	0	0	21	54	25

Supplementary Table 4. Ranking possibilities of ORR

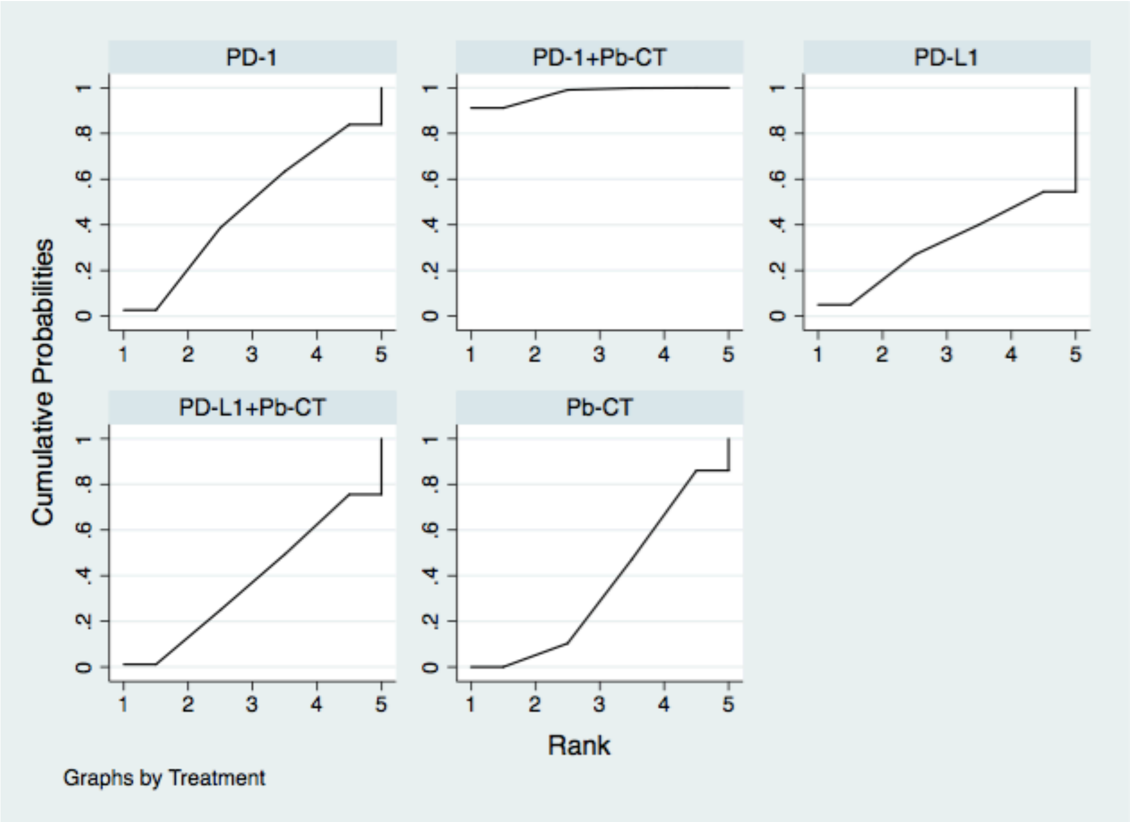

Supplementary Figure 23. SUCRA ranking plot of ORR

Treatment	SUCRA	PrBest	MeanRank
PD-1	47.2	2.7	3.1
PD-L1	31.6	5.0	3.7
PD-1+Pb-CT	97.5	91.2	1.1
PD-L1+Pb-CT	37.8	1.1	3.5
Pb-CT	35.9	0.0	3.6

Supplementary Figure 24. SUCRA ranking possibilities of ORR

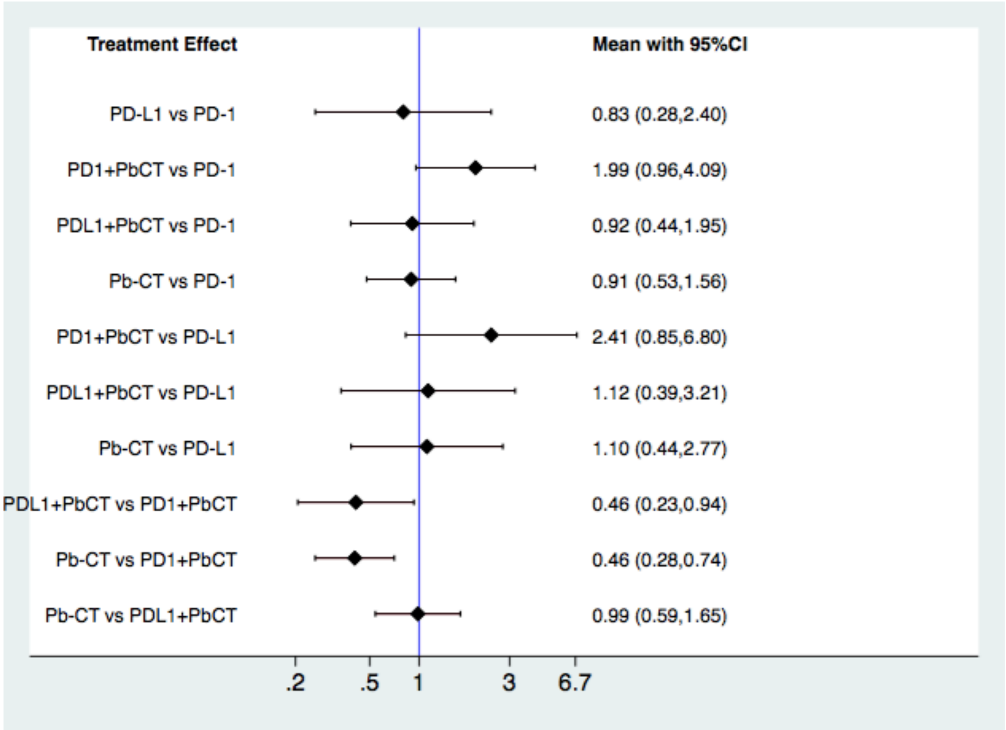

Supplementary Figure 25. Pairwise meta-analysis forest plot for ORR

(Note: Pb-CT=platinum-based chemotherapy)

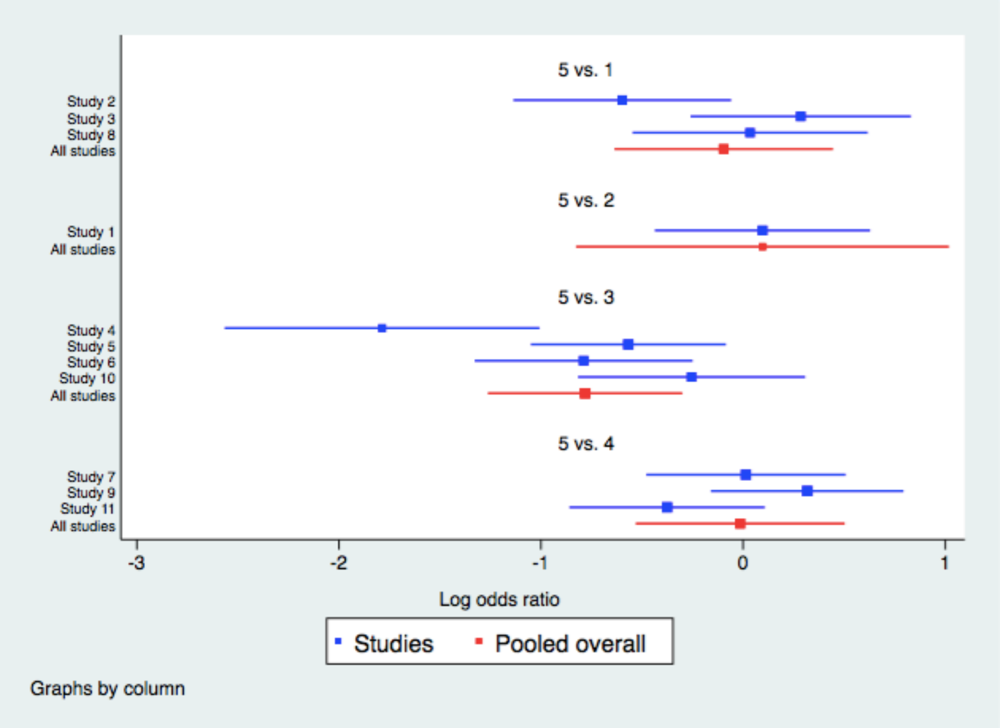

Supplementary Figure 26. Consistency analysis forest plot of ORR

(NOTE: Study 1-11 was **IMpower110, KEYNOTE024, CheckMate026, KEYNOTE021, KEYNOTE407-1, KEYNOTE407-2, IMpower131, KEYNOTE042, IMpower130, KEYNOTE189, IMpower132)**

```
Side     Direct                   Indirect                 Difference
>    tau
         Coef.       Std. Err.    Coef.       Std. Err.    Coef.       Std. Err.   P>|z|
A E *    -.0959272   .2755476     -.2396706   27.49807     .1437434    27.49946    0.996
>     .3842506
B E *     .0958453   .4705858     -.1900949   202.1557     .2859402    202.1563    0.999
>     .3842448
C E *    -.7817252   .2452452     -.1675786   70.85198     -.6141466   70.85243    0.993
>     .3842493
D E *    -.0141548   .2637162     -.2031425   96.71401     .1889877     96.7144    0.998
>     .3842466
```

Supplementary Figure 27. Node-split plot for ORR (all p-value >0.05 (consistency))

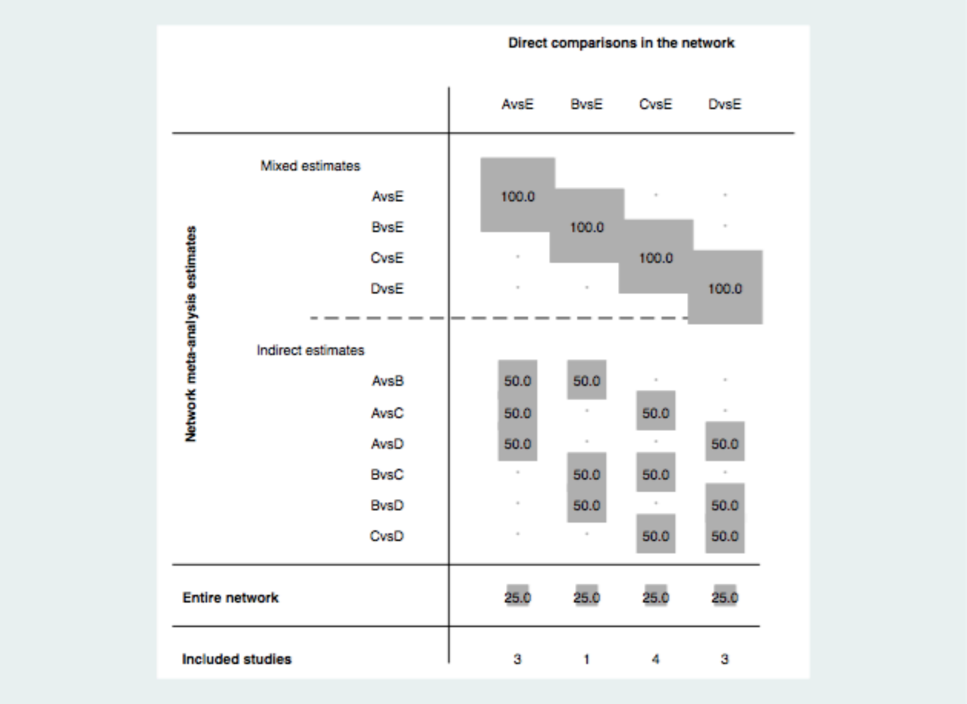

Supplementary Figure 28. Contribution plot of trials for ORR

(**A**=**PD-1, B=PD-L1, C=PD-1+Pb-CT, D=PD-L1+Pb-CT, E=Pb-CT**)

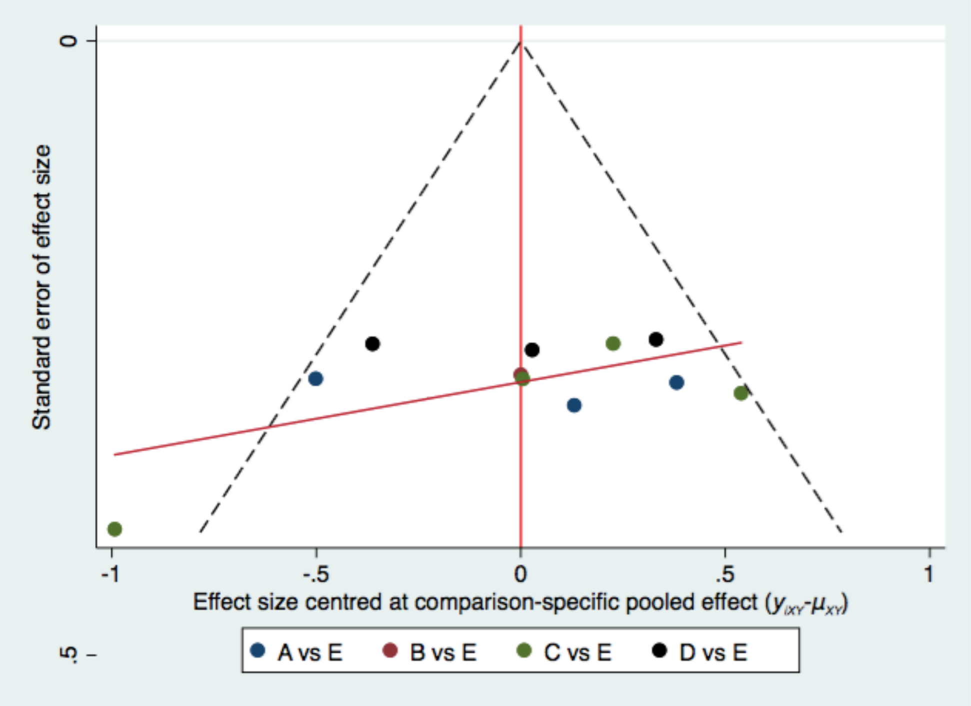

Supplementary Figure 29. Funnel plot of trials for ORR

(**A**=**PD-1, B=PD-L1, C=PD-1+Pb-CT, D=PD-L1+Pb-CT, E=Pb-CT**)

9. Adverse events

All adverse events

Grade 3-5 adverse events

**PD-1**	2.47 (0.75 to 7.32)	7.73 (2.99 to 19.88)	12.22 (4.69 to 34.36)	4.77 (2.68 to 7.75)
0.69 (0.14 to 1.35)	**PD-L1**	3.15 (0.90 to 11.63)	4.96 (1.39 to 20.34)	1.94 (0.72 to 5.44)
0.22 (0.13 to 0.34)	0.32 (0.16 to 0.62)	**PD-1+Pb-CT**	1.60 (0.51 to 5.20)	0.62 (0.27 to 1.35)
0.14 (0.08 to 0.24)	0.21 (0.10 to 0.44)	0.66 (0.39 to 1.14)	**PD-L1+Pb-CT**	0.39 (0.15 to 0.88)
0.28 (0.19 to 0.39)	0.40 (0.22 to 0.74)	1.28 (0.93 to 1.80)	1.93 (1.28 to 2.97)	**Pb-CT**

Supplementary Figure 30. Pooled estimates for all kinds of adverse events

(Note: Pb-CT=platinum-based chemotherapy)

All diarrhea

Grade 3-5 diarrhea

**PD-1**	…	1.73 (0.93 to 3.19)	1.66 (0.73 to 3.53)	1.10 (0.70 to 1.68)
…	**PD-L1**	…	…	…
1.14 (0.24 to 7.33)	…	**PD-1+Pb-CT**	0.96 (0.43 to 2.03)	0.64 (0.41 to 0.97)
1.81 (0.25 to 13.69)	…	1.59 (0.19 to 10.43)	**PD-L1+Pb-CT**	0.67 (0.35 to 1.27)
1.86 (0.57 to 6.44)	…	1.62 (0.45 to 4.95)	1.02 (0.21 to 5.28)	**Pb-CT**

Supplementary Figure 31. Pooled estimates for treatment-related diarrhea

(Note: Pb-CT=platinum-based chemotherapy)

All fatigue

Grade 3-5 fatigue

**PD-1**	…	2.97 (1.34 to 8.34)	2.44 (0.70 to 9.18)	2.48 (1.30 to 4.91)
…	**PD-L1**	…	…	…
0.16 (0.02 to 0.76)	…	**PD-1+Pb-CT**	0.82 (0.21 to 2.81)	0.83 (0.39 to 1.49)
0.26 (0.03 to 2.31)	…	1.64 (0.22 to 18.04)	**PD-L1+Pb-CT**	1.01 (0.33 to 3.03)
0.27 (0.07 to 0.90)	…	1.69 (0.57 to 7.45)	1.03 (0.17 to 6.22)	**Pb-CT**

Supplementary Figure 32. Pooled estimates for treatment-related fatigue

(Note: Pb-CT=platinum-based chemotherapy)

All decreasing appetite

Grade 3-5 decreasing appetite

**PD-1**	…	2.79 (1.62 to 5.10)	3.72 (1.77 to 8.20)	3.14 (2.12 to 4.73)
…	**PD-L1**	…	…	…
0.19 (0.01 to 1.92)	…	**PD-1+Pb-CT**	1.35 (0.61 to 2.87)	1.13 (0.73 to 1.62)
0.25 (0.01 to 5.67)	…	1.34 (0.05 to 59.76)	**PD-L1+Pb-CT**	0.84 (0.44 to 1.60)
0.23 (0.02 to 1.16)	…	1.24 (0.19 to 12.96)	0.93 (0.06 to 15.50)	**Pb-CT**

Supplementary Figure 33. Pooled estimates for treatment-related decreasing appetite

(Note: Pb-CT=platinum-based chemotherapy)

All anemia (upper triangle); Grade 3-5 anemia (lower triangle)

**PD-1**	…	11.65 (3.78 to 36.33)	17.14 (3.80 to 84.89)	13.93 (6.57 to 34.77)
…	**PD-L1**	…	…	…
0.05 (0.01 to 0.15)	…	**PD-1+Pb-CT**	1.46 (0.34 to 7.30)	1.19 (0.59 to 2.82)
0.02 (0.00 to 0.11)	…	0.52 (0.11 to 2.54)	**PD-L1+Pb-CT**	0.81 (0.21 to 3.16)
0.04 (0.01 to 0.10)	…	0.86 (0.37 to 1.94)	1.65 (0.42 to 6.15)	**Pb-CT**

Supplementary Figure 34. Pooled estimates for treatment-related anemia

(Note: Pb-CT=platinum-based chemotherapy)

All nausea (upper triangle); Grade 3-5 nausea (lower triangle)

**PD-1**	…	9.43 (5.59 to 16.82)	8.51 (4.09 to 17.35)	7.65 (5.15 to 11.42)
…	**PD-L1**	…	…	…
0.03 (0.00 to 0.46)	…	**PD-1+Pb-CT**	0.90 (0.43 to 1.77)	0.81 (0.54 to 1.16)
0.02 (0.00 to 0.62)	…	0.67 (0.02 to 32.02)	**PD-L1+Pb-CT**	0.91 (0.50 to 1.65)
0.03 (0.00 to 0.34)	…	1.14 (0.20 to 11.86)	1.71 (0.09 to 39.86)	**Pb-CT**

Supplementary Figure 35. Pooled estimates for treatment-related nausea

(Note: Pb-CT=platinum-based chemotherapy)

**PD-1**	0.41 (0.12 to 1.46)	1.47 (0.24 to 11.06)	0.11 (0.04 to 0.30)
2.43 (0.68 to 8.50)	**PD-1+CT**	3.57 (0.68 to 24.47)	0.26 (0.12 to 0.53)
0.68 (0.09 to 4.09)	0.28 (0.04 to 1.47)	**PD-L1+Pb-CT**	0.07 (0.01 to 0.32)
9.29 (3.39 to 26.22)	3.83 (1.89 to 8.15)	13.59 (3.17 to 81.18)	**Pb-CT**

Supplementary Figure 36. Pooled estimates for ICB-related hypothyroidism

(Note: Pb-CT=platinum-based chemotherapy)

**PD-1**	0.38 (0.04 to 3.75)	1.84 (0.06 to 161.83)	0.11 (0.02 to 0.64)
2.62 (0.27 to 22.96)	**PD-1+CT**	4.82 (0.19 to 291.17)	0.29 (0.07 to 1.01)
0.54 (0.01 to 17.20)	0.21 (0.00 to 5.24)	**PD-L1+Pb-CT**	0.06 (0.00 to 1.08)
9.08 (1.57 to 57.11)	3.48 (0.99 to 13.50)	16.57 (0.93 to 907.56)	**Pb-CT**

Supplementary Figure 37. Pooled estimates for ICB-related hyperthyroidism

(Note: Pb-CT=platinum-based chemotherapy)

**PD-1**	0.18 (0.03 to 1.41)	0.32 (0.03 to 3.82)	0.06 (0.01 to 0.29)
5.61 (0.71 to 34.60)	**PD-1+CT**	1.74 (0.17 to 13.44)	0.33 (0.10 to 0.83)
3.15 (0.26 to 37.74)	0.57 (0.07 to 5.76)	**PD-L1+Pb-CT**	0.19 (0.03 to 1.20)
17.11 (3.40 to 89.74)	3.05 (1.21 to 10.28)	5.32 (0.83 to 35.27)	**Pb-CT**

Supplementary Figure 38. Pooled estimates for ICB-related pneumonitis

(Note: Pb-CT=platinum-based chemotherapy)

**PD-1**	0.10 (0.00 to 1.45)	0.06 (0.00 to 0.52)
9.65 (0.69 to 274.90)	**PD-1+Pb-CT**	0.62 (0.12 to 2.62)
15.67 (1.92 to 312.73)	1.62 (0.38 to 8.63)	**Pb-CT**

Supplementary Figure 39. Pooled estimates for ICB-related skin reaction

(Note: Pb-CT=platinum-based chemotherapy)

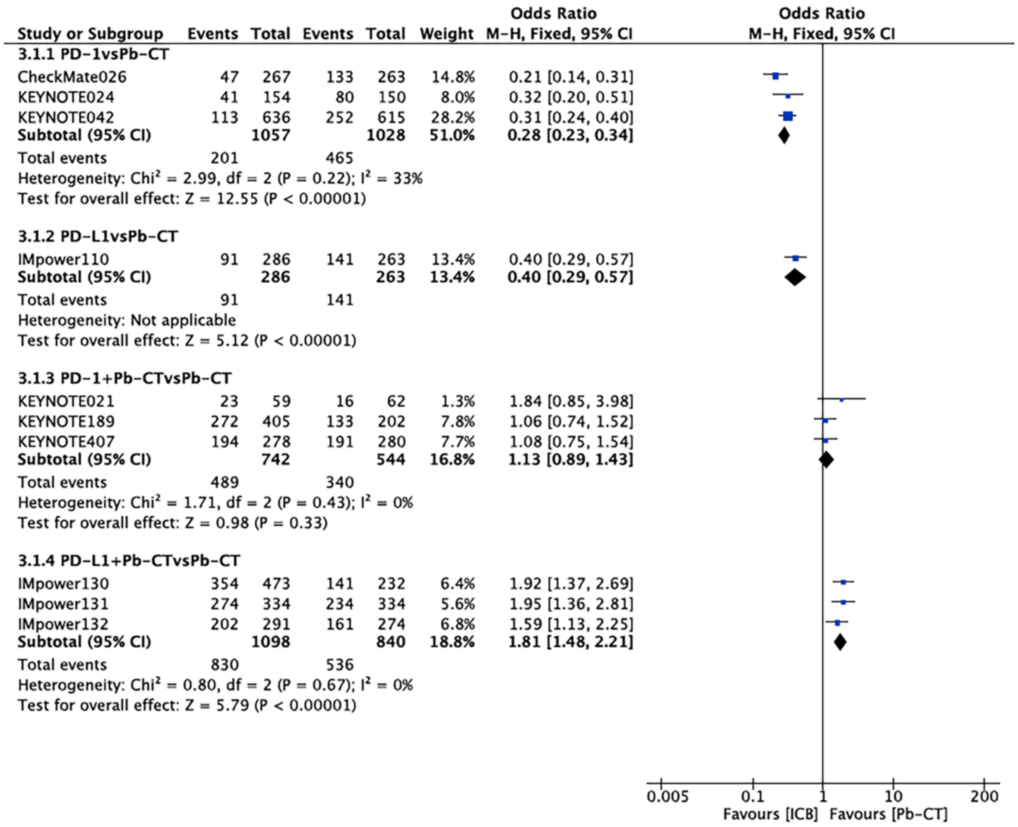

Supplementary Figure 40. Pairwise meta-analysis for grade 3-5 adverse events

(Note: Pb-CT=platinum-based chemotherapy)

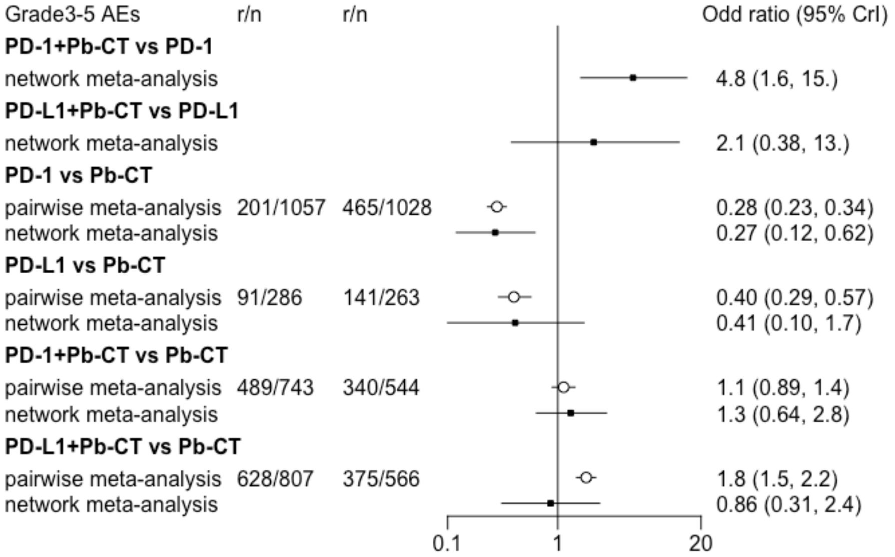

Supplementary Figure 41. Direct and indirect meta-analysis for grade 3-5 adverse events

(Note: Pb-CT=platinum-based chemotherapy)

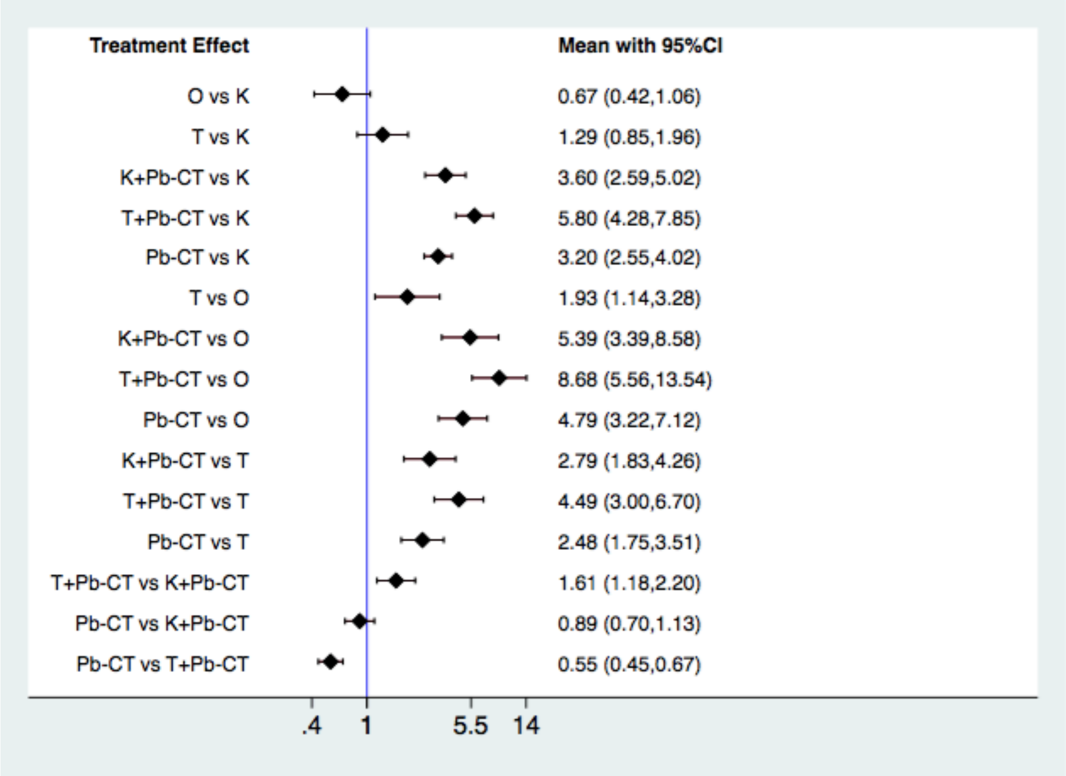

Supplementary Figure 42. Pairwise analysis forest plot for sever adverse events (grade 3-5)

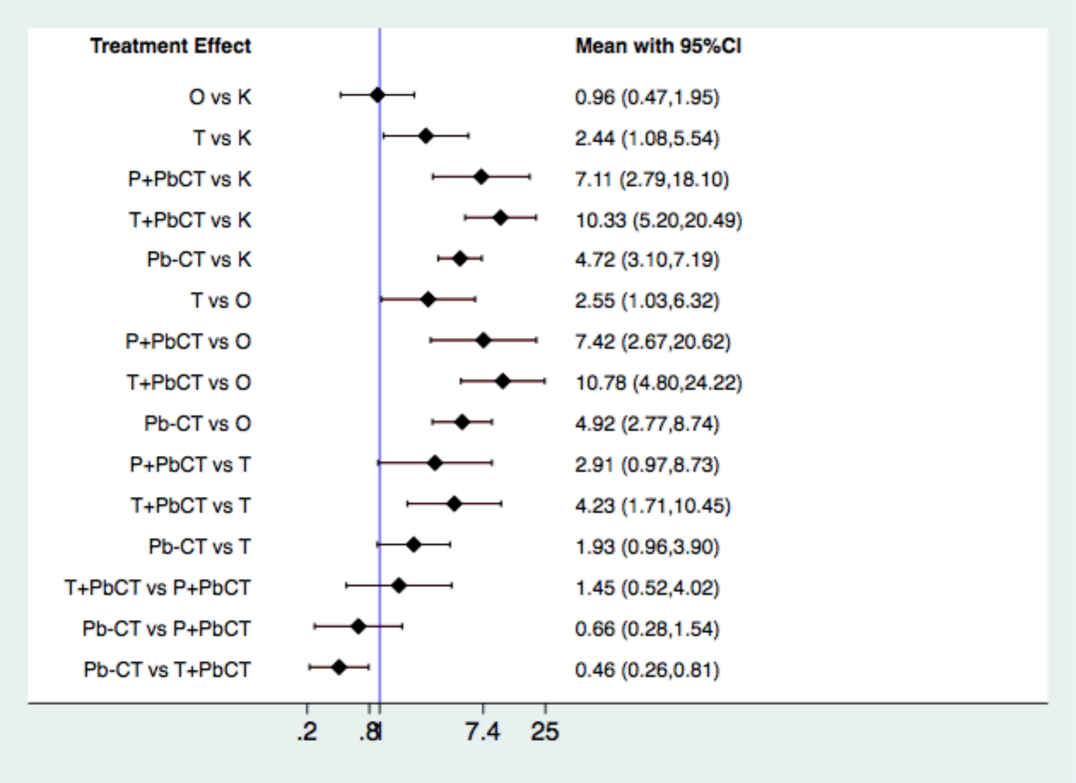

Supplementary Figure 43. Pairwise analysis forest plot for all adverse events (grade 1-5)

(K=Pembrolizumab; O=Nivolumab; T=Atezolizumab)

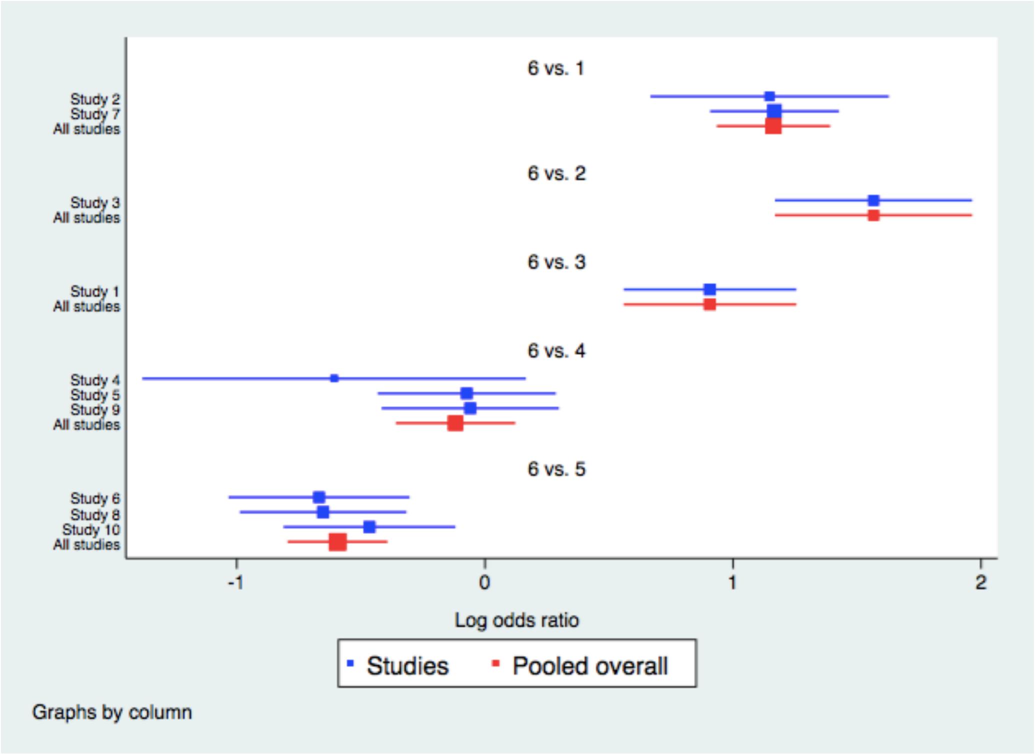

Supplementary Figure 44. Consistency analysis forest plot of server AEs (grade 3-5)

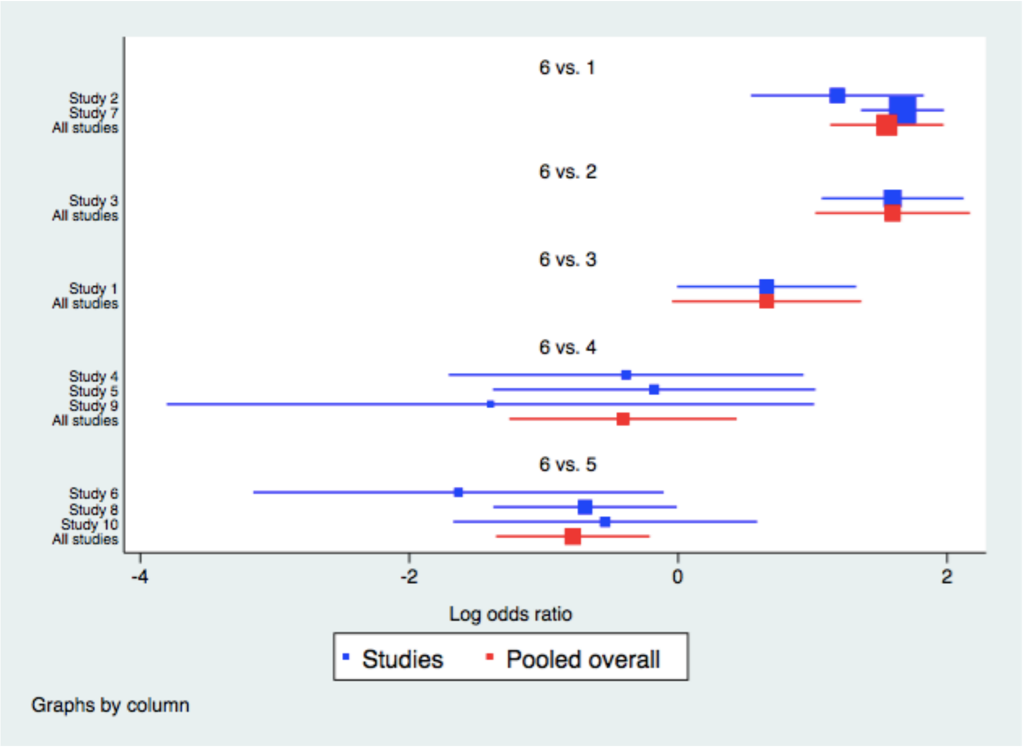

Supplementary Figure 45. Consistency analysis forest plot of all AEs (grade 1-5)

(NOTE: Study 1-10 was **IMpower110, KEYNOTE024, CheckMate026, KEYNOTE021, KEYNOTE407, IMpower131, KEYNOTE042, IMpower130, KEYNOTE189, IMpower132)**

```
        Coef.      Std. Err.  Coef.      Std. Err.  Coef.      Std. Err.  P>|z|
A F *    1.551402    .214642   .2987537   49.53205   1.252648   49.53247  0.980
>    .1155236
B F *    1.594111    .292771   2.694156      163.5  -1.100045   163.5005  0.995
>    .1155398
C F *    .6576405   .3583074   2.985308   238.0407  -2.327667   238.0411  0.992
>    .1155393
D F *    -.410528   .4313388    3.07933   199.6331  -3.489858   199.6334  0.986
>    .1155353
E F *   -.7834333   .2913276   3.142941   214.0989  -3.926374   214.0989  0.985
>    .1155357
```

Supplementary Figure 46. Node-split plot for severe AEs

```
Side    Direct                Indirect              Difference
>   tau
        Coef.      Std. Err.  Coef.      Std. Err.  Coef.      Std. Err.  P>|z|
A F *    1.162737   .1163637   .0157157     23.948   1.147021   23.94828  0.962
>    1.11e-08
B F *    1.566295   .2025664   2.516152   133.8848  -.9498572   133.8849  0.994
>    3.62e-10
C F *    .9068789   .1772178   2.318493    127.774  -1.411614   127.7741  0.991
>    8.23e-09
D F *   -.1187685   .1227492   2.330436   78.06095  -2.449204   78.06107  0.975
>    7.80e-09
E F *   -.5943909   .1025806   2.368338   80.11552  -2.962729   80.11559  0.971
>    1.09e-08
```

Supplementary Figure 47. Node-split plot for all AEs

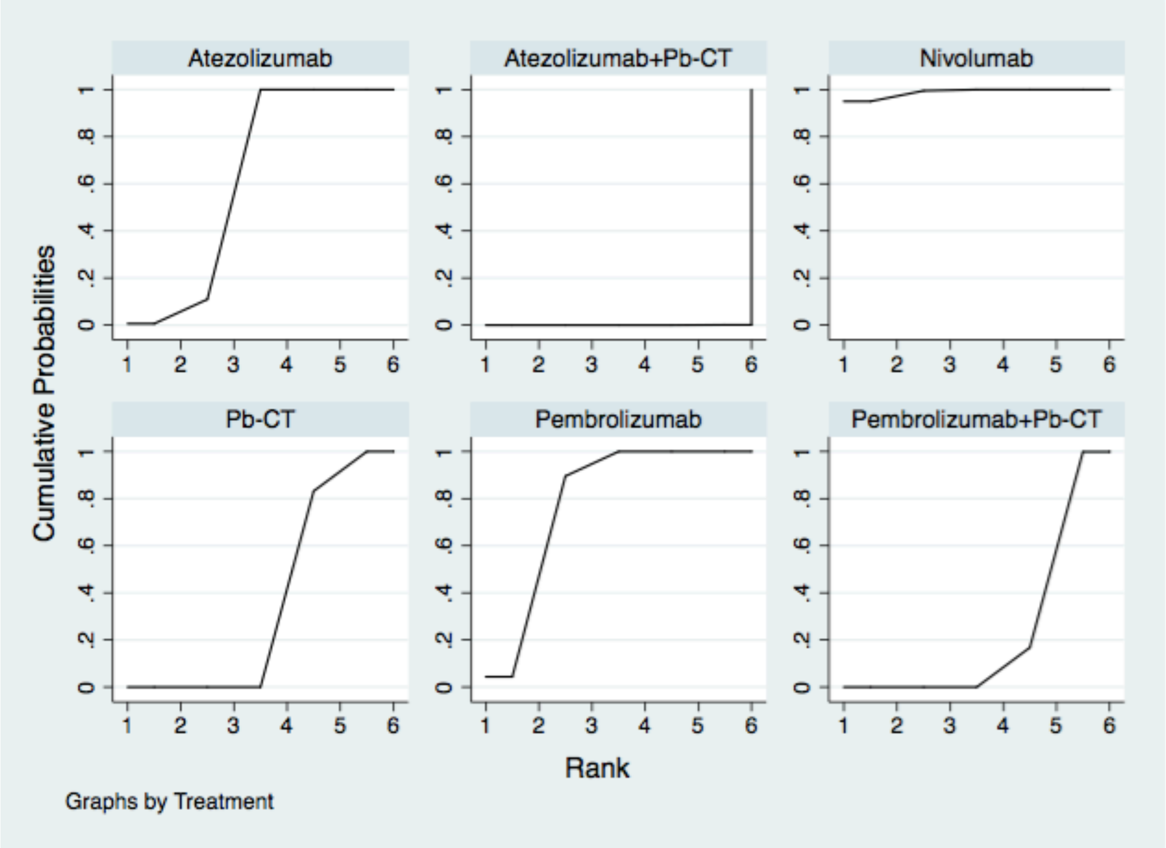

Supplementary Figure 48. SUCRA ranking plot of severe AEs (grade 3-5) (**min to max**)

Treatment	SUCRA	PrBest	MeanRank
Pembrolizumab	78.8	4.4	2.1
Nivolumab	98.9	95.0	1.1
Atezolizumab	62.3	0.6	2.9
Pembrolizumab+Pb-CT	23.3	0.0	4.8
Atezolizumab+Pb-CT	0.0	0.0	6.0
Pb-CT	36.6	0.0	4.2

Supplementary Figure 49. SUCRA ranking plot of severe AEs (grade 3-5) (**min to max**)

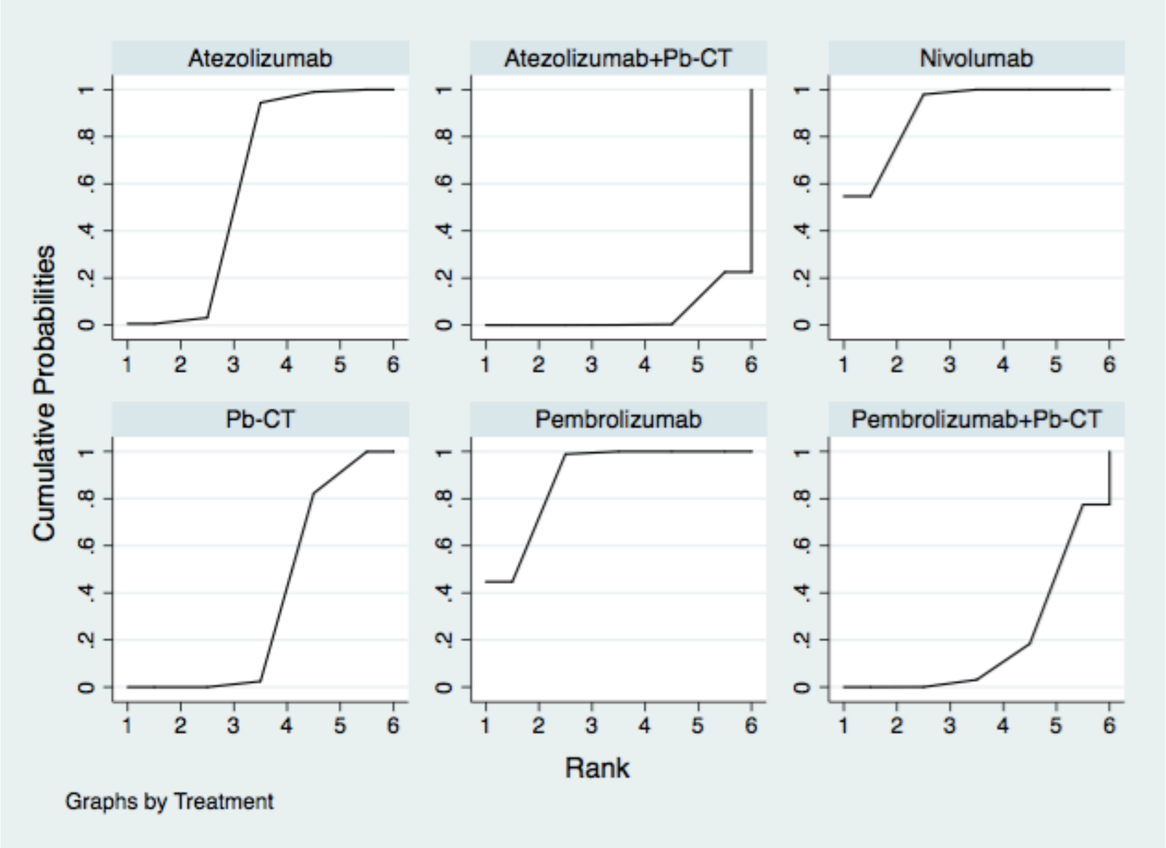

Supplementary Figure 50. SUCRA ranking plot of severe AEs (grade 1-5) (**min to max**)

Treatment	SUCRA	PrBest	MeanRank
Pembrolizumab	88.7	44.7	1.6
Nivolumab	90.5	54.7	1.5
Atezolizumab	59.4	0.6	3.0
Pembrolizumab+Pb-CT	19.8	0.0	5.0
Atezolizumab+Pb-CT	4.6	0.0	5.8
Pb-CT	36.9	0.0	4.2

Supplementary Figure 51. SUCRA ranking plot of severe AEs (grade 1-5) (**min to max**)

(Note: Pb-CT=platinum-based chemotherapy)

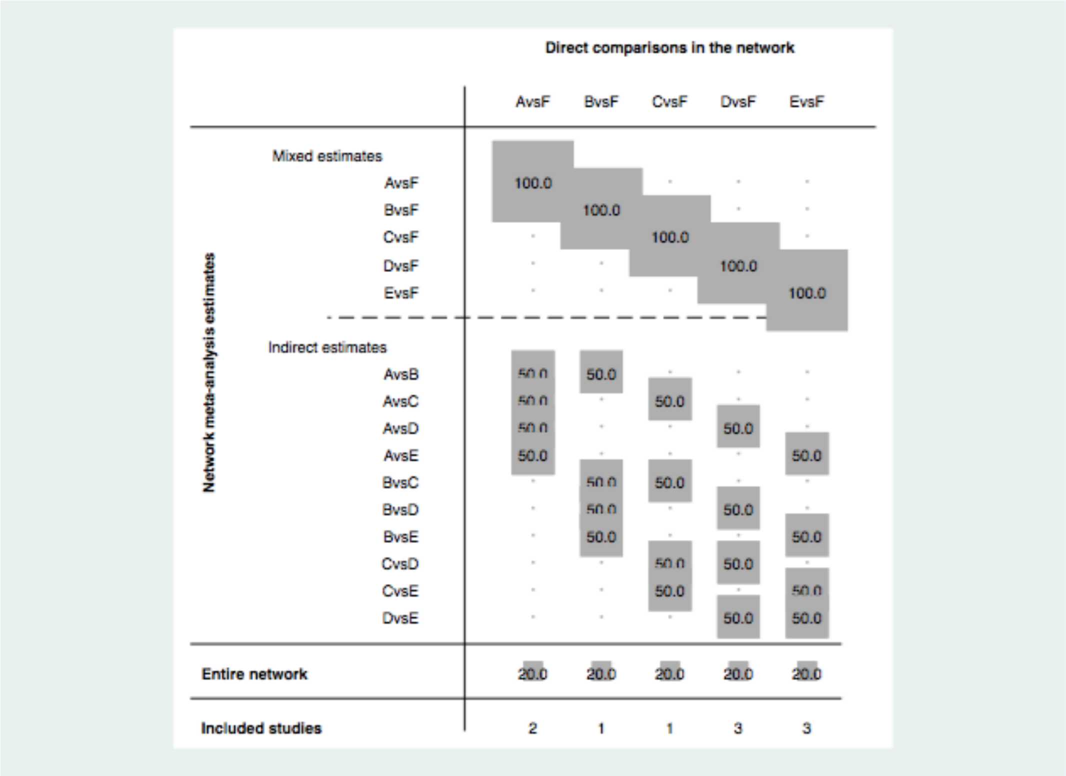

Supplementary Figure 52. Contribution plot of the included trials

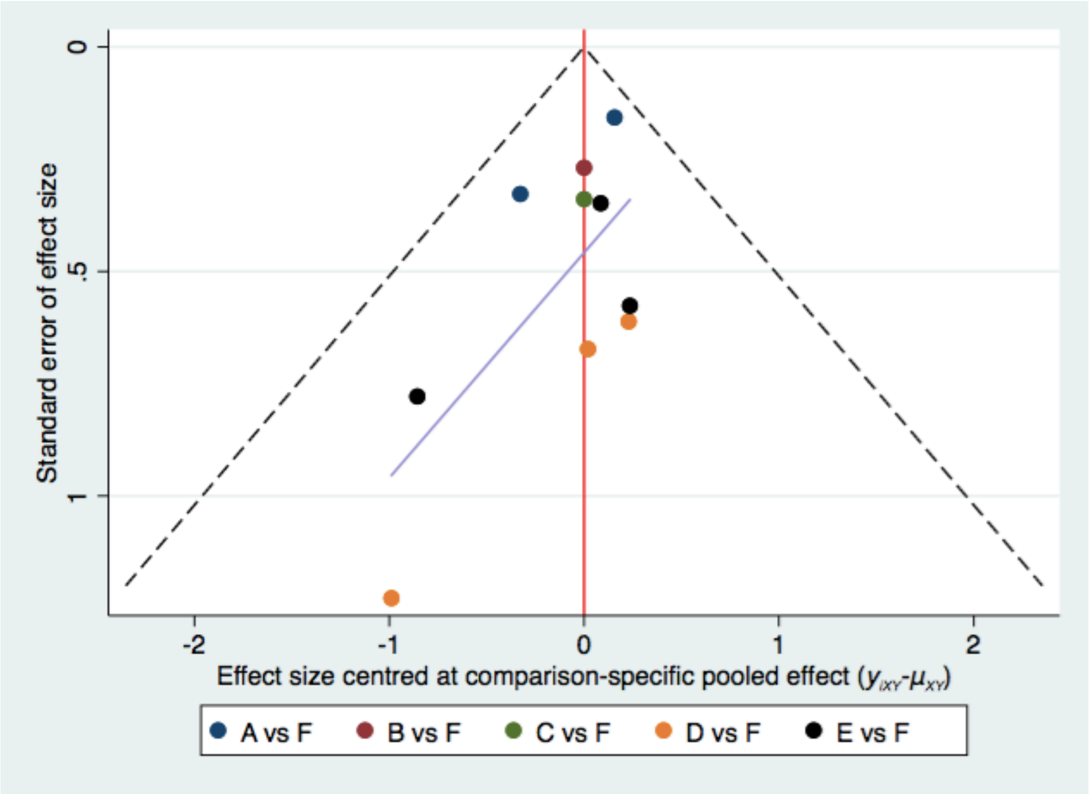

Supplementary Figure 53. Funnel plot of the included trials

(A=PD-1, B=PD-L1, C=PD-1+Pb-CT, D=PD-L1+Pb-CT, E=Pb-CT)
